# Elucidating the Structures of Substituted Adamantyl Esters and Ethers Using Rotational Spectroscopy and Computations

**DOI:** 10.1002/cphc.202500035

**Published:** 2025-06-05

**Authors:** Nataša Burić, Donatella Loru, Jasna Alić, Marina Šekutor, Melanie Schnell, Pablo Pinacho

**Affiliations:** ^1^ Department of Organic Chemistry and Biochemistry Ruđer Bošković Institute Bijenička cesta 54 10000 Zagreb Croatia; ^2^ Deutsches Elektronen‐Synchrotron DESY Notkestr. 85 22607 Hamburg Germany; ^3^ Christian‐Albrechts‐Universität zu Kiel Institute of Physical Chemistry Max‐Eyth‐Str. 1 24118 Kiel Germany; ^4^ Department of Physical Chemistry and Inorganic Chemistry IU‐CINQUIMA University of Valladolid Paseo Belen 7 47011 Valladolid Spain

**Keywords:** adamantyl derivatives, increasing size substituents, molecular structures, rotational spectroscopy

## Abstract

Adamantane derivatives are promising candidates in the design of new materials with unique properties. In this study, the molecular structure of a series of adamantyl esters and ethers with an increasing substituent size using broadband rotational spectroscopy is investigated. The experimental structure for three of the compounds using different methods to compare them with theoretical bond distances and angles is determined. The influence on oxygen functional group variation as well as the increasing size of the second alkyl substituent on the adopted gas‐phase structure is assessed. This study advances previous work on similar systems to shine more light on the molecular features of adamantyl covalent assemblies with oxygen atoms.

## Graphical abstract

Five adamantyl esters and ethers with alkyl substituents of increasing branching are investigated by rotational spectroscopy. From the experimental data, their accurate structures are derived. All the derivatives feature similar C—C distances, regardless of the functional group, the alkyl substituent, or other effects as methyl top internal rotation, confirming the robustness and practical importance of diamondoid cages in molecular design.

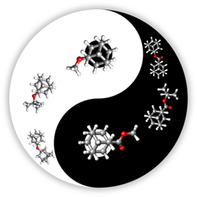

## 1 Introduction

Diamondoids are bulky cage hydrocarbons of different sizes and high symmetries that structurally resemble the diamond crystal structure at the molecular scale.^[^ ^1^ ^]^ Adamantane is the smallest diamondoid, and it consists of 10 carbon and 16 hydrogen atoms arranged as three fused cyclohexane rings. Such a unique shape imparts high structural stability to the cage itself as well as to adamantane‐containing derivatives, which arises from the lack of strain compared to other alkanes.^[^ ^1^, ^2^, ^3^, ^4^, ^5^ ^]^

Diamondoids are often used as building blocks in the design of new complex molecules and materials with desired properties. While the adamantyl subunit conveys bulkiness and lipophilicity, its functionalization and further build‐up with other groups modulate the physical and chemical properties of the formed covalent assemblies.^[^ ^1^, ^6^ ^]^ Those compounds have increasingly attracted attention due to their application in medicinal chemistry. Adamantane derivatives have already been proven as excellent drugs against viral or bacterial infections or even against the effects of Parkinson's disease.^[^ ^3^ ^]^ In addition, the shape and lipophilicity of adamantane in such compounds allow for more specific targeting in drug delivery.^[^ ^3^, ^6^, ^7^, ^8^, ^9^ ^]^

As the old saying goes, “form follows function”, so to design new materials with specific properties, it is necessary to have a detailed description of the molecular structure to later investigate the relationship between the structure and the biological activity. The introduction of diverse functional groups and substituents can affect the structure of adamantyl derivatives, resulting in a modulation of their physical, chemical, and biological properties. To characterize this effect, the molecular structure can be studied using different spectroscopic techniques. Parent adamantane, along with several of its derivatives, have been studied by nuclear magnetic resonance (NMR),^[^ ^10^ ^]^ infrared spectroscopy (IR),^[^ ^11^, ^12^ ^]^ electronic photodissociation spectroscopy,^[^ ^13^, ^14^ ^]^ X‐ray and gas‐phase electron diffraction,^[^ ^15^ ^]^ and rotational spectroscopy.^[^ ^16^, ^17^, ^18^ ^]^

The latter is a prime technique for obtaining experimental molecular structures with high accuracy.^[^ ^19^ ^]^ Current spectrometers work in a broadband manner, recording a broad range of the spectrum in a single acquisition. Averaging millions of acquisitions in a short time improves the signal‐to‐noise ratio, while the high resolution inherent to the technique allows for distinguishing between isomers, conformers, and even isotopologues.^[^ ^20^, ^21^, ^22^ ^]^ One of the most powerful features of rotational spectroscopy is the ability to detect isotopologues. By analyzing the rotational spectra of monosubstituted isotopologues, often in their natural abundance, it is possible to derive experimental structures by determining the bond distances and angles in a molecule. These experimental molecular structures can then be used to understand the physical, chemical, and biological properties of the system.

**Figure 1:**
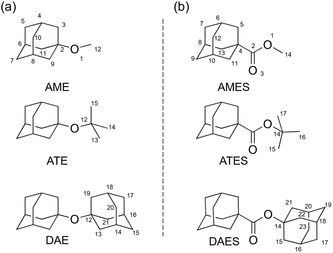
a) Structures of the ether derivatives: 1‐adamantyl methyl ether (AME), 1‐adamantyl *tert*‐butyl ether (ATE), and 1,1'‐diadamantyl ether (DAE, already reported).^[^ ^16^ ^]^ b) Structures of the ester derivatives: 1‐adamantyl methyl ester (AMES), 1‐adamantyl *tert*‐butyl ester (ATES), and 1,1'‐diadamantyl ester (DAES). The complete atom labeling is exemplified in the structures of AME and AMES; in the other structures, only the different part is labeled.

Previous studies using rotational spectroscopy on adamantyl derivatives included adamantan‐2‐ol,^[^ ^23^ ^]^ 1,1‐diadamantyl ether (DAE),^[^ ^16^, ^17^ ^]^ and a series of adamantyl thioethers with increasing alkyl substituent size.^[^ ^18^ ^]^ In these studies, we reported on the experimental structure of the adamantyl group in different environments, together with an analysis of the interplay between hydrogen bonding and dispersion interactions. In this work, we investigated five previously unexplored adamantyl derivatives using rotational spectroscopy. We describe efficient synthetic routes for obtaining these derivatives, along with their accurate structures. The molecules studied are adamantyl ethers and esters with increasingly branched alkyl substituents (**Figure** **1**), thus extending the existing structural knowledge on such covalent assemblies constructed using different oxygen‐containing functional groups.^[^ ^16^, ^18^ ^]^

## 2 Results and Discussion

The molecules studied in this work are depicted in Figure 1. 1‐Adamantyl methyl ether (C_11_H_18_O, AME) and 1‐adamantyl *tert*‐butyl ether (C_14_H_24_O, ATE) complete our envisioned series of cage ethers with different substituents, together with the already described 1,1'‐diadamantyl ether (C_20_H_30_O, DAE).^[^ ^16^ ^]^ They feature a methyl and a *tert*‐butyl group, respectively, attached to the bulky adamantane moiety through the ether oxygen atom. Note that this series is complementary to the recently reported adamantyl thioethers,^[^ ^18^ ^]^ that contained a sulfur atom instead of an oxygen. In this work, we additionally studied a hitherto unexplored adamantyl ester series of compounds, guided by the increasing substituent size principle. Thus, we prepared 1‐adamantyl methyl ester (C_12_H_18_O_2_, AMES), 1‐adamantyl *tert*‐butyl ester (C_15_H_24_O_2_, ATES), and 1,1'‐diadamantyl ester (C_21_H_30_O_2_, DAES). Note that in these structures, the carboxylic group is anchored to the adamantane cage, while the hydroxyl oxygen of the ester is connected to a second alkyl substituent.

**Figure 2:**
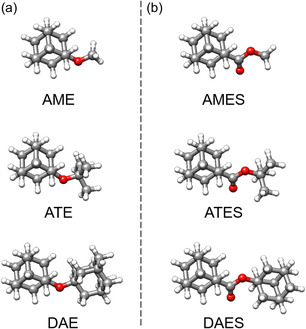
3D depiction of a) ether derivatives, AME, ATE, and DAE,^[^ ^16^ ^]^ and b) the ester counterparts, AMES, ATES, and DAES.

The five molecules are quite rigid and only present one conformation, simplifying the assignment of their rotational spectra. The spectra of the five molecules, except for ATE, are dominated by *b*‐type rotational transitions, aligning well with their predicted values for the dipole‐moment components (**Table** **1** and **2**, Figure S1, Supporting Information). The observed geometries of the five molecules exhibit an interesting symmetry (**Figure** **2**). For all of them, except for ATE, the alkyl substituent aligns in the center of the plane formed by the adamantyl group (Figure S2, Supporting Information). In the spectra of AME and AMES, some splittings due to the internal rotation of the methyl top attached to either the ether or ester oxygen atom are observed. The fitting of the splitting allowed for the determination of the *V*_3_ barrier height of the motion (*vide infra*).

**Table 1:** Experimental and theoretical (B3LYP‐D3(BJ)/def2‐TZVP) spectroscopic constants for AME and ATE. The final fit for AME was performed with XIAM, while SPFIT was used for ATE (see text for details).

	AME		ATE	
	Experimental	Theoretical	Experimental	Theoretical
*A* [MHz]a)	1624.96977(86)b)	1631.3	1229.14479(19)	1232.9
*B* [MHz]	845.73805(38)	845.6	417.963170(97)	418.7
*C* [MHz]	834.75871(38)	834.4	417.892195(96)	418.6
Δ_J_ [kHz]	[0.0]c)	–	0.00352(68)	–
Δ_JK_ [kHz]	0.105(11)	–	[0.0]	–
Δ_K_ [kHz]	[0.0]	–	[0.0]	–
*δ*_J_ [kHz]	[0.0]	–	[0.0]	–
*δ*_K_ [kHz]	[0.0]	–	[0.0]	–
*D*_π2J_ [MHz]	0.289(21)	–	–	–
*D*_π2K_ [MHz]	−0.626(29)	–	–	–
*D*_π2‐_ [MHz]	0.318(23)	–	–	–
*V*_3_ [cm^−1^]	389.81(12)	510	–	–
*V*_3_ [kJ mol^−1^]	4.6632(15)	6.1	–	–
*μ*_a_/*μ*_b_/*μ*_c_ [D]	y/y/n	0.7/−1.1/0.0	y/y/y	0.3/0.4/1.0
*N*	149	–	241	–
*σ* [kHz]	16.4	–	4.5	–

a) *A*, *B*, and *C* are the rotational constants; Δ_J_, Δ_JK_, Δ_K,_ *δ*_J_, and *δ*_K_ are the quartic centrifugal distortion constants. *D*_π2J_, *D*_π2K_, and *D*_π2‐_ are internal rotation‐overall rotation distortion tunneling parameters. *V*_3_ is the barrier height for the internal rotation of the methyl top. *μ*_a_, *μ*_b_, and *μ*_c_ are the electric dipole‐moment components of the transitions (n: not observed, y: observed). *N* is the number of fitted transitions. *σ* is the root‐mean‐square deviation of the fit.

b) Standard error is in parentheses in units of the last digit.

c) Parameters in square brackets were kept fixed to zero.

**Table 2:** Experimental and theoretical (B3LYP‐D3(BJ)/def2‐TZVP) spectroscopic constants for AMES, ATES, and DAES. The final fit for AMES was performed with XIAM, while SPFIT was used for ATES and DAES (see text for details).

	AMES		ATES		DAES	
	Experimental	Theoretical	Experimental	Theoretical	Experimental	Theoretical
*A* [MHz]a)	1420.36697(56)^b)^	1423.6	1054.68846(23)	1055.1	753.4267(11)	754.2
*B* [MHz]	520.86840(30)	521.0	290.628679(71)	290.5	136.82353(30)	136.8
*C* [MHz]	494.54033(30)	494.1	280.036130(73)	279.7	134.28930(41)	134.2
Δ_J_ [kHz]	0.0113(23)	–	[0.0]	–	[0.0]	–
Δ_JK_ [kHz]	0.0179(49)	–	0.0143(17)	–	[0.0]	–
Δ_K_ [kHz]	[0.0]^c)^	–	[0.0]	–	0.127(30)	–
*δ*_J_ [kHz]	[0.0]	–	[0.0]	–	[0.0]	–
*δ*_K_ [kHz]	−0.177(46)	–	[0.0]	–	[0.0]	–
*D*_π2J_ [MHz]	0.1519(47)	–	–	–	–	–
*D*_π2K_ [MHz]	−0.595(10)	–	–	–	–	–
*D*_π2‐_ [MHz]	0.1389(55)	–	–	–	–	–
*V*_3_ [cm^−1^]	325.078(33)	300	–	–	–	–
*V*_3_ [kJ mol^−1^]	3.88880(39)	3.6	–	–	–	–
*μ*_a_ */* *μ*_b_/*μ*_c_	y/y/n	−0.5/1.8/0.0	n/y/n	0.1/−1.9/0.0	n/y/n	0.5/−1.8/0.0
*N*	268	–	143	–	35	–
*σ* [kHz]	15.4	–	7.3	–	7.5	–

a) See Table 1 for parameter definition.

b) Standard error is in parentheses in units of the last digit.

c) Parameters in square brackets were kept fixed to zero.

By detecting the ^13^C monosubstituted isotopologues in natural abundance (≈1.1% abundance of ^12^C) of ATE, AMES, and ATES (Tables S1–S3, Supporting Information), their experimental structures could be determined. The intensities of the rotational transitions in the spectra of AME and DAES were not sufficient to detect ^13^C isotopologue signals, preventing the determination of the experimental structures. However, considering the good agreement between the experimental and the theoretical parameters (Table 1 and 2), it is reasonable to assume that the theoretical structures of AME and DAES are close enough to the experimental ones.

**Figure 3:**
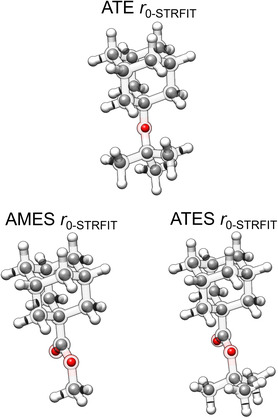
Experimental (*r*_0_, inner solid spheres) structure obtained with STRFIT overlaid with the theoretical (*r*_e_, outer ball and stick semitransparent model) structure from computations.

Two methods have been used in this work to determine the molecular structures from the experimental parameters, namely the *r*_s_ and the *r*_0_ approaches. Both rely on the availability of the experimental rotational constants for each isotopologue. In the first one, the Kraitchman equations are solved^[^ ^24^ ^]^ to obtain the atomic coordinates in the inertial axis system of the molecule. This method does not rely on any input from theory to determine the structure; however, it has several drawbacks. If the mono‐substituted isotopologue for an atom is not detected, it is not possible to obtain its coordinates. Additionally, if any of the atoms are close to a principal inertial axis or plane, it can give an imaginary value of the respective atomic coordinate. Finally, this method only yields the absolute values of the atomic coordinates, and therefore, signs should be assigned from theoretical calculations. We did not detect ^18^O or ^2^H isotopologues for any of the studied molecules, meaning that we could only determine the positions of the carbon atoms in the molecular frame. Furthermore, in the case of ATES, one of the ^13^C isotopologues could not be identified in the spectrum, thus resulting in an incomplete *r*_s_ structure. These problems can be overcome using the *r*_0_ method, in which the structure is obtained by performing a least‐squares fit of selected bond distances and angles. The parameters are chosen to achieve the best agreement with the rotational constants of the molecule in the vibrational ground state, giving the *r*_0_ structure.^[^ ^25^ ^]^ For a more detailed comparison, we used two programs, which employ different approaches to determine the *r*_0_ structure: STRFIT^[^ ^26^ ^]^ and UNEX.^[^ ^27^ ^]^ Both programs give complementary information by performing a different determination of the ground‐state geometry. The first one obtains the *r*_0_ structure by a least‐squares fit of selected bond distances, angles, and dihedral angles. The second controls the amount of experimental and theoretical information in the final structure by varying one parameter. As in previous studies of similar adamantyl compounds,^[^ ^16^, ^18^ ^]^ due to the rigidity of the molecules, adding terms to account for molecular vibrations^[^ ^28^ ^]^ does not improve the structural fit. **Figure** **3** presents a superposition of the *r*_0_ and the *r*_e_ structure for ATE, AMES, and ATES. The complete set of results from both *r*_s_ and *r*_0_, along with the theoretical *r*_e_ structures, is collected in Tables S4–S9 and Figures S3–S5, Supporting Information.

The differences between ethers and esters originate from the presence of a carbonyl group in the esters. However, it is worth noting that in adamantane derivatives, both functional groups exhibit a similar arrangement and alignment of the alkyl substituents (Figure S2, Supporting Information). Experimental data demonstrate that the C—C bond distances within the adamantyl group are around 1.54 Å for the different derivatives, which is a typical value for a C—C single bond. A subtle difference can be observed in the distances involving the heteroatoms between ether and ester derivatives, originating from their different nature. However, both functional groups present consistency in the O1—C2 and O1—C12/13 bond distances regardless of the substituent (Table S10, Supporting Information). The C—C—C angles in the adamantyl group are ≈109.5°, mirroring those of isolated adamantane.^[^ ^1^ ^]^ This structural similarity demonstrates the preservation of the adamantyl frame and the alkyl substituent position while providing some flexibility in selecting the functional group. This adaptability is a benefit for the design of new functional materials, as it implies that the adamantane subunit conserves its structure and arrangement while allowing for diverse functionalization.

In two spectra, those for AME and AMES, some of the rotational transitions exhibited splittings. As in the thioether analogue, this splitting was attributed to the internal rotation of the methyl top attached either to the ether or to the ester. To estimate the barrier height, a scan of the internal rotation motion was performed by varying the H–C–O–C dihedral angle using computations at the B3LYP‐D3(BJ)/def2‐TZVP level of theory. The predicted *V*_3_ barriers (Figure S6, Supporting Information) were used as starting values for the analysis of the splitting, resulting in the fit of *V*_3_. Floating *ρ* (dimensionless internal rotation vector) or *β*, the angle between *ρ* and the principal inertial axis *a*, did not improve the fit, although it was needed to include additional terms for the internal rotation–overall rotation distortion (*D*_π2K_, *D*_π2J_, and *D*_π2‐_). The experimental *V*_3_ values 389.81(12) and 325.078(33) cm^−1^ for AME and AMES, respectively, are slightly smaller than other O–CH_3_ tops,^[^ ^29^, ^30^ ^]^ but in the same range as in methyl carbamate.^[^ ^31^ ^]^ Curiously, the barrier for methyl top internal rotation in AME compared to AMES is reduced by around 15% in the ester derivative. However, the chemical environment and the symmetry in the vicinity of the methyl top are similar in both molecules (Figure S6, Supporting Information). Interestingly,^[^ ^18^ ^]^ the barrier for the internal rotation of the methyl group in AME is slightly smaller than the one for the sulfur analog (AMT), opposite to the tendency from other O/S compounds analyzed.^[^ ^29^ ^]^

## 3 Conclusions

We investigated five previously unexplored ethers and esters featuring an adamantyl group combined with alkyl substituents of increasing branching using high‐resolution rotational spectroscopy to characterize their molecular structures. The studied molecules are rather rigid, with only one conformation predicted in the gas phase, resulting in relatively symmetric structures. Due to the observation of the ^13^C isotopologues spectra, it was possible to derive the experimental structure for three out of the five derivatives following the *r*_s_ and *r*_0_ methodologies. The bond distances and angles determined experimentally show an excellent agreement with those predicted by quantum‐chemical computations, thus advancing our knowledge of variously substituted adamantyl derivatives in the gas phase. Two of the molecules studied present splittings of the rotational transitions due to the internal rotation of a methyl top. In one case, the methyl top is attached to the ether and, in the other, to the ester, allowing us to explore the differences in the barrier height depending on the functional group. Since the adamantyl group is present in numerous compounds with fascinating properties, accurately describing how its core molecular structure is affected in different environments is crucial for understanding their biological roles or material building block assembly capabilities. Our findings show that even though esters and ethers differ chemically, in the adamantyl derivatives these functional groups share enough geometric similarities to result in the same alignment of the alkyl group. The combination of stability in the alkyl parts and versatility in the functionalization makes adamantane derivatives valuable compounds in the design of materials with specific properties and reactivity.

## 4 Experimental Section and Computational Details

4.1

4.1.1

##### Materials and Methods

All ^1^H and ^13^C NMR spectra were recorded with Bruker AV‐300 or AV‐600 NMR spectrometers, and the NMR spectra were referenced to the residual proton or carbon signal of the used deuterated solvent as an internal standard. IR spectra were recorded with a Fourier transform infrared (FT‐IR) ABB Bomem MB 102 spectrometer (range 400–4000 cm^−1^). Matrix‐assisted laser desorption/ionization time‐of‐flight mass spectrometry (MALDI‐TOF MS) results were obtained in “reflectron” mode with an Applied Biosystems Voyager DE STR instrument (Foster City, CA). Gas chromatography‐mass spectrometry (GC‐MS) analyses were performed on an Agilent 7890B/5977B GC/MSD instrument equipped with a HP‐5 ms column. Determination of melting points was performed using an Original Kofler Mikroheitztisch apparatus (Reichert, Wien). All the solvents, 1‐adamantylcarboxylic acid, and adamantan‐1‐ol were obtained from commercial sources and used without further purification. 1,1'‐Diadamantyl ether (DAE)^[^ ^16^ ^]^ and 1‐adamantyl‐*tert*‐butyl ether (ATE)^[^ ^32^ ^]^ were prepared according to our previously published procedures.

##### 1‐Adamantyl‐Methyl Ether (AME)

1‐Adamantyl methanesulfonate^[^ ^16^ ^]^ (1.15 g, 5 mmol), absolute methanol (5 mL), and TEA (0.84 mL, 6 mmol) were heated under nitrogen at 60 °C for 24 h. The reaction mixture was cooled to room temperature and transferred to a separating funnel, and the flask was additionally washed with dichloromethane (3 × 5 mL). The organic phase was washed with water (3 × 5 mL), dried over Na_2_SO_4_, and filtered, and the solvent was removed by a rotary evaporator without heating the water bath. The resulting crude product was purified by column chromatography (SiO_2_, *n*‐hexane as eluent), yielding AME^[^ ^33^ ^]^ (623 mg, 75%) as a colorless viscous liquid possessing a pleasant smell. ^1^H NMR (600 MHz, CDCl_3_), *δ*/ppm: 3.22 (s, 3 H), 2.14 (br s, 3 H), 1.73–1.71 (m, 6 H), 1.66–1.56 (m, 6 H). ^13^C NMR (150 MHz, CDCl_3_), *δ*/ppm: 71.9 (C), 47.7 (CH, 3C), 40.9 (CH_2_, 3C), 36.5 (CH_2_, 3C), 30.5 (CH_3_) (Figure S7, Supporting Information). IR (NaCl plates), ${\tilde{\nu }}_{\text{max}}/{\text{cm}}^{-1}$: 2909 (br s), 2853 (m), 1638 (w), 1453 (m), 1354 (m), 1305 (w), 1199 (m), 1091 (s), 1053 (m), 893 (m), 747 (w). MS (EI), *m*/*z*: 166.2 (M^+^).

##### Methyl‐1‐Adamantanecarboxylate (AMES)

A suspension of dried MgSO_4_ (722 mg, 6 mmol) and concentrated H_2_SO_4_ (110 μL, 2 mmol) in methanol (25 mL) was stirred at RT for 20 min, and then 1‐adamantylcarboxylic acid (270 mg, 1.5 mmol) was added. The reaction mixture was stirred under reflux for 16 h. After cooling of the reaction mixture, solid dry MgSO_4_ (361 mg, 3 mmol) and solid dry Na_2_CO_3_ (636 mg, 6 mmol) were added, and after stirring for 20 min at room temperature, the salts were filtered off under vacuum using a sinter funnel (porosity 4). The solvent from the filtrate was evaporated, and the remaining crude product was purified by column chromatography (SiO_2_, dichloromethane as eluent), yielding ester AMES^[^ ^34^ ^]^ (267 mg, 91%) as a viscous oil that solidified upon standing in the air at room temperature, giving white crystals. M.p. 39–40 °C (lit.^[^ ^35^ ^]^ 37–39 °C). ^1^H NMR (300 MHz, CDCl_3_), *δ*/ppm: 3.64 (s, 3 H), 1.98–2.03 (m, 3 H), 1.88 (d, *J* = 2.8 Hz, 6 H), 1.65–1.76 (m, 6 H). ^13^C NMR (75 MHz, CDCl_3_), *δ*/ppm: 178.2 (C, 1C), 51.5 (OCH_3_, 1C), 40.7 (C, 1C), 38.9 (CH_2_, 3C), 36.5 (CH_2_, 3C), 27.9 (CH, 3C) (Figure S8, Supporting Information). IR (KBr), ${\tilde{\nu }}_{\text{max}}/{\text{cm}}^{-1}$: 2907 (br s), 2853 (s), 1732 (s), 1453 (m), 1425 (m), 1323 (w), 1269 (m), 1238 (s), 1184 (m), 1103 (m), 1076 (s), 993 (w), 965 (w), 878 (w), 740 (w), 6275 (w). MS (EI), *m*/*z*: 194.2 (M^+^).

##### *Tert*‐Butyl‐1‐Adamantanecarboxylate (ATES)

A suspension of dried MgSO_4_ (2.6 g, 21.6 mmol) and concentrated H_2_SO_4_ (350 μL, 6.8 mmol) in dry dichloromethane (30 mL) was stirred in a reaction tube at RT for 20 min. 1‐Adamantylcarboxylic acid (1.1 g, 6.1 mmol) dissolved in dry dichloromethane (10 mL) was added, and the reaction mixture was cooled to –10 °C with an ice salt bath (ice‐NaCl‐acetone). Then *tert*‐butanol (1.7 g, 22.9 mmol) dissolved in dry dichloromethane (8 mL) was added, the reaction tube was immediately sealed with a Teflon stopper, and the mixture was stirred on an ice salt bath for 2 h and afterward at RT for 2 days.^[^ ^36^ ^]^ The reaction mixture was transferred to a separating funnel, and the flask was additionally washed with dichloromethane (3 × 5 mL). The organic phase was washed with water (3 × 30 mL) and dried over Na_2_SO_4_, filtered, and the solvent was evaporated. The remaining crude product was purified by column chromatography (SiO_2_, dichloromethane as eluent), yielding ester ATES^[^ ^37^ ^]^ (930 mg, 53%) as a white solid. M.p. 44–46 °C (lit.^[^ ^38^ ^]^ 42–44 °C). ^1^H NMR (300 MHz, CDCl_3_), *δ*/ppm: 1.95–2.03 (m, 3 H), 1.84 (d, *J *= 2.7 Hz, 6 H), 1.65–1.73 (m, 6 H), 1.42 (s, 9 H). ^13^C NMR (75 MHz, CDCl_3_), *δ*/ppm: 177.2 (C=O, 1C), 79.3 (C(CH_3_)_3_, 1C), 41.1 (C, 1C), 38.9 (CH_2_, 3C), 36.6 (CH_2_, 3C), 28.1 (CH or CH_3_, 3C), 28.0 (CH or CH_3_, 3C) (Figure S9, Supporting Information). IR (KBr), ${\tilde{\nu }}_{\text{max}}/{\text{cm}}^{-1}$: 2904 (br s), 2852 (s), 1712 (s), 1478 (w), 1455 (w), 1367 (w), 1325 (w), 1271 (m), 1250 (m), 1166 (m), 1105 (w), 1080 (m), 976 (w), 910 (w), 852 (w), 810 (w), 771 (w), 742 (w). MS (EI), *m*/*z*: 181.1.

##### 1‐Adamantyl‐1‐Adamantanecarboxylate (DAES)

A suspension of dried MgSO_4_ (722 mg, 6 mmol) and concentrated H_2_SO_4_ (110 μL, 2 mmol) in 1,2‐dichloroethane (20 mL) was stirred at RT for 20 min, and then 1‐adamantylcarboxylic acid (270 mg, 1.5 mmol) and adamantan‐1‐ol (685 mg, 4.5 mmol) were added. The reaction mixture was stirred under reflux for 16 h. After cooling of the reaction mixture, solid dry MgSO_4_ (361 mg, 3 mmol) and solid dry Na_2_CO_3_ (636 mg, 6 mmol) were added, and after stirring for 20 min at room temperature, the salts were filtered off under vacuum using a sinter funnel (porosity 4). The solvent from the filtrate was evaporated, and the remaining crude product was purified by column chromatography (SiO_2_, dichloromethane as eluent), yielding ester DAES^[^ ^39^ ^]^ (470 mg, 99%) as a white solid. M.p. 277–278 °C. ^1^H NMR (600 MHz, CDCl_3_), *δ*/ppm: 2.14 (br. s, 3 H), 2.08 (d, 6 H, *J *= 2.4 Hz), 1.99 (br. s, 3 H), 1.83 (d, 6 H, *J *= 2.2 Hz), 1.62–1.73 (m, 12 H). ^13^C NMR (150 MHz, CDCl_3_), *δ*/ppm: 177.0 (C, 1C), 79.3 (C, 1C), 41.3 (CH_2_, 3C), 41.1 (C, 1C), 38.9 (CH_2_, 3C), 36.6 (CH_2_, 3C), 36.3 (CH_2_, 3C), 30.8 (CH, 3C), 28.1 (CH, 3C) (Figure S10, Supporting Information). IR (KBr), ${\tilde{\nu }}_{\text{max}}/{\text{cm}}^{-1}$: 2908 (s), 2849 (sm), 1742 (wm), 1725 (s), 1234 (s), 1078 (sm), 1058 (m). MS (EI), *m*/*z*: 270.3 for [M–CO_2_]^+^. HRMS (MALDI): calcd. for [C_21_H_30_O_2_+Na]^+^ 337.2143; found 337.2140.

##### Chirped‐Pulse Fourier‐Transform Microwave (CP‐FTMW) Spectroscopy

The spectra for the five derivatives were recorded using the COMPACT spectrometer in Hamburg^[^ ^21^ ^]^ based on broadband chirped‐pulse Fourier‐transform microwave (CP‐FTMW) spectroscopy.^[^ ^40^ ^]^ The spectra were recorded between 2 and 8 GHz of frequency with slightly different conditions for each of the compounds. The samples were held in an internal reservoir and heated (**Table** **3**) to bring enough of the molecules into the gas phase. The gas was diluted in Ne at a backing pressure of around 2 bar and introduced into the vacuum chamber by a pulsed valve, at a repetition rate of 8 Hz. A chirped pulse covering the 2‐8 GHz frequency region with 4 *μ*s duration was generated, amplified, and broadcasted into the chamber to induce the macroscopic polarization of the ensemble. Once the radiation ceased, the free‐induction decay (FID) signal from the polarized molecular ensemble was recorded in the time domain and Fourier transformed. A fast‐frame setup^[^ ^41^ ^]^ allowed to record eight emission–detection cycles per supersonic expansion, resulting in a repetition rate of 64 Hz. The intensities of the spectra of some compunds were sufficient to detect signals arising from the monoisotopic substitution with ^13^C in natural abundance (≈1.1% of ^12^C), allowing to determine the experimental structures using the *r*_s_ and *r*_0_ methods. The spectrometer featured a resolution between nearby lines better than 25 kHz and accuracy in frequency determination better than 15 kHz.

**Table 3:** Experimental conditions for the five molecules reported in this work, including the heating temperature of the sample, the number of averaged acquisitions (# FIDs), and the detection or not of ^13^C isotopologues.

Molecule	Heating [ºC]	# FIDs	^13^C
AME	35	1 600 000	No
ATE	50	4 400 000	Yes
AMES	65	4 800 000	Yes
ATES	65	4 100 000	Yes
DAES	180	700 000	No

The spectra were analyzed with the program JB95^[^ ^42^ ^]^ to obtain preliminary fits for each molecule and with Pickett´s program suite CALPGM^[^ ^43^ ^]^ to obtain the final fits using a semirigid rotor Hamiltonian in the A‐reduction and I^r^ representation.^[^ ^19^, ^44^ ^]^ For the two molecules with methyl‐top internal rotation, the final fits were obtained with XIAM.^[^ ^45^ ^]^ The complete line lists of fitted transitions are given in the supplementary information file in Tables S11–S15, Supporting Information.

##### Computational Details

Due to the rigidity of the molecules studied here, only a few rotamers were expected. Their conformational landscape was explored using the CREST code^[^ ^46^ ^]^ implementing GFN2‐xTB.^[^ ^47^, ^48^ ^]^ A geometry optimization for each monomer was performed at the B3LYP‐D3(BJ)/def2‐TZVP^[^ ^49^, ^50^, ^51^ ^]^ level of theory, within the harmonic approximation to obtain their zero‐point corrected relative energies. Dihedral angle scans were performed to predict the barrier height for the methyl top internal rotation at the B3LYP‐D3(BJ)/def2‐TZVP level of theory. All computations were done using the program package Orca 5.0.^[^ ^52^, ^53^ ^]^

## Conflict of Interest

The authors declare no Conflict of interest.

## Supporting information

Supplementary Material

## Data Availability

The data that support the findings of this study are available from the corresponding author upon reasonable request.
