# Supplementary material for: Elucidating the Structures of Substituted Adamantyl Esters and Ethers Using Rotational Spectroscopy and Computations
Source: Chemphyschem. 2025 Jun 5;26(15):e202500035. doi: 10.1002/cphc.202500035 (PMC12321282; doi:10.1002/cphc.202500035)
Supplement: Supplementary file 1 — Supplementary Material [file CPHC-26-e202500035-s001.pdf]

Supplementary information for:

Elucidating the structures of substituted adamantyl esters and ethers using rotational spectroscopy and computations

Nataša Burić,[1,+] Donatella Loru,[2,+] Jasna Alić,[1] Marina Šekutor,[1,*] Melanie Schnell,[2,3,*] Pablo Pinacho[2,4*]

[1] Department of Organic Chemistry and Biochemistry, Ruđer Bošković Institute, Bijenička cesta 54, 10000 Zagreb, Croatia.

[2] Deutsches Elektronen-Synchrotron DESY, Notkestr. 85, 22607 Hamburg, Germany.

[3] Christian-Albrechts-Universität zu Kiel, Institute of Physical Chemistry Max-Eyth-Str. 1, 24118 Kiel, Germany.

[4] Department of Physical Chemistry and Inorganic Chemistry, IU-CINQUIMA, University of Valladolid, Paseo Belen 7, 47011 Valladolid, Spain.

[+] Shared first authorship.

Table of Contents:

Figure S1. Zoom-in of the rotational spectra.
Figure S2. Alternative views for the molecules studied in this work along with DAE.
Figure S3. Overlay of the experimental (r_s and r_0) and theoretical (r_e) structures for ATE.
Figure S4. Overlay of the experimental (r_s and r_0) and theoretical (r_e) structures for AMES.
Figure S5. Overlay of the experimental (r_s and r_0) and theoretical (r_e) structures for ATES.
Figure S6. V_3 barrier for AME and AMES.
Figure S7. NMR spectra of AME.
Figure S8. NMR spectra of AMES.
Figure S9. NMR spectra of ATES.
Figure S10. NMR spectra of DAES.
Table S1. Rotational constants for ATE for the parent and its observed isotopologues.
Table S2. Rotational constants for AMES for the parent and its observed isotopologues.
Table S3. Rotational constants for ATES for the parent and its observed isotopologues.
Table S4. Atomic coordinates from the experimental and theoretical structures for ATE.
Table S5. Structural parameters from the experimental and theoretical structures for ATE.
Table S6. Atomic coordinates from the experimental and theoretical structures for AMES.
Table S7. Structural parameters from the experimental and theoretical structures for AMES.
Table S8. Atomic coordinates from the experimental and theoretical structures for ATES.
Table S9. Structural parameters from the experimental and theoretical structures for ATES.
Table S10. Comparison of experimental and theoretical selected bond distances.
Table S11. Observed frequencies for the parent species of AME.
Table S12. Observed frequencies for the parent species of ATE.
Table S13. Observed frequencies for the isotopologues of ATE.
Table S14. Observed frequencies for the parent species of AMES.
Table S15. Observed frequencies for the isotopologues of AMES.
Table S16. Observed frequencies for the parent species of ATES.
Table S17. Observed frequencies for the isotopologues of ATES.
Table S18. Observed frequencies for the parent species of DAES.
Table S19. Cartesian coordinates from the theoretical computation for AME.
Table S20. Cartesian coordinates from the theoretical computation for ATE.
Table S21. Cartesian coordinates from the theoretical computation for AMES.
Table S22. Cartesian coordinates from the theoretical computation for ATES.
Table S23. Cartesian coordinates from the theoretical computation for DAES.

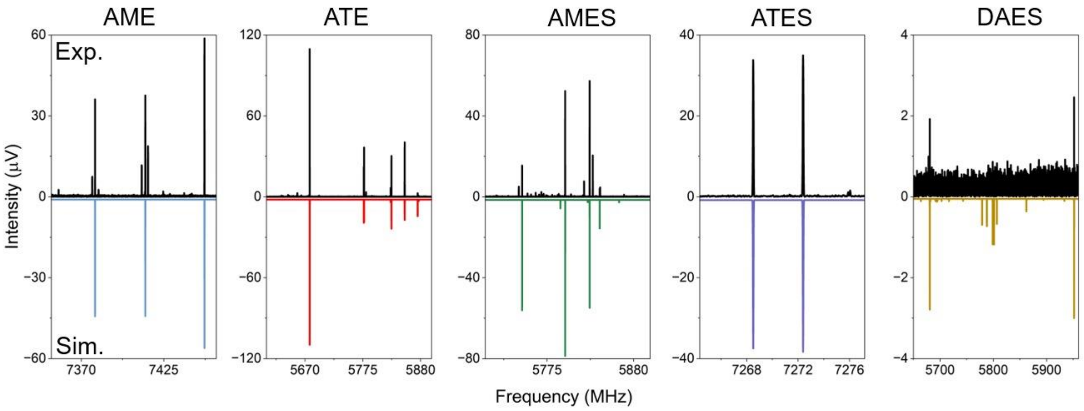

Figure S1. Zoom-in of the rotational spectra showing selected transitions. The upward traces (black) are the experimental spectra, and the downward traces (blue for AME, red for ATE, green for AMES, purple for ATES, and yellow for DAES) are simulations based on the fitted constants.

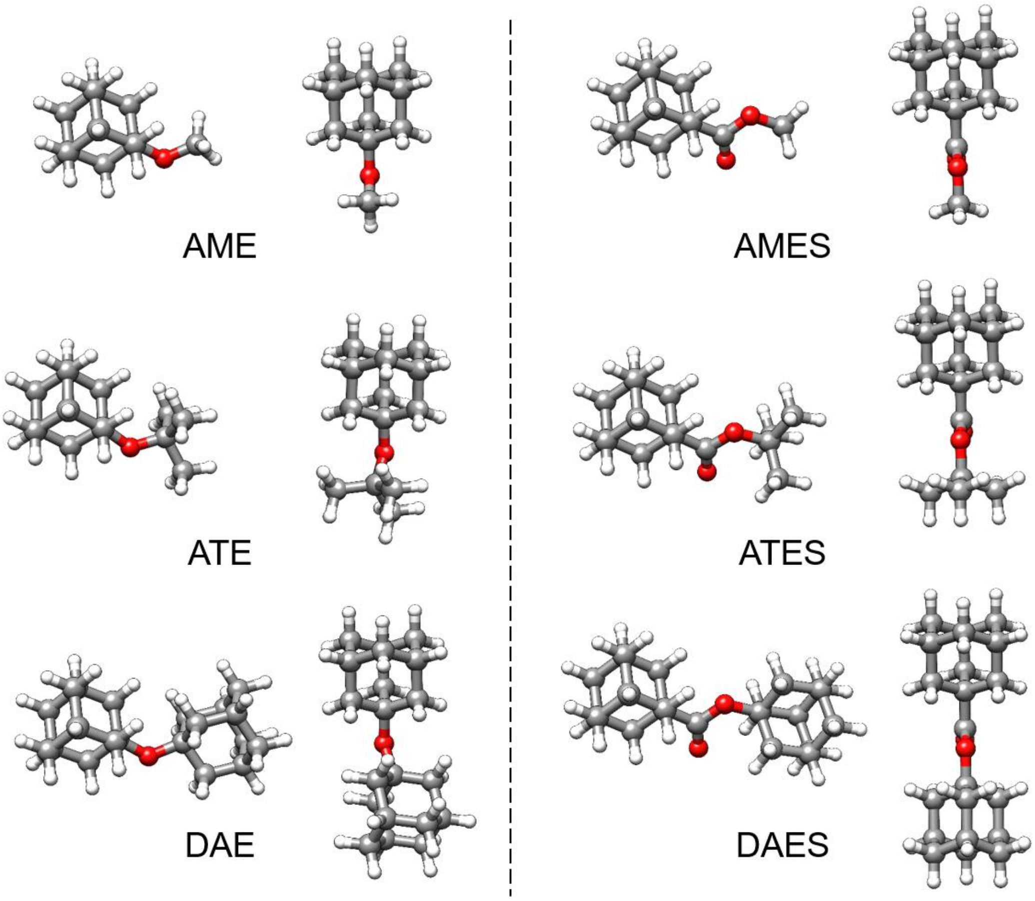

Figure S2. Alternative views for the molecules studied in this work along with DAE.

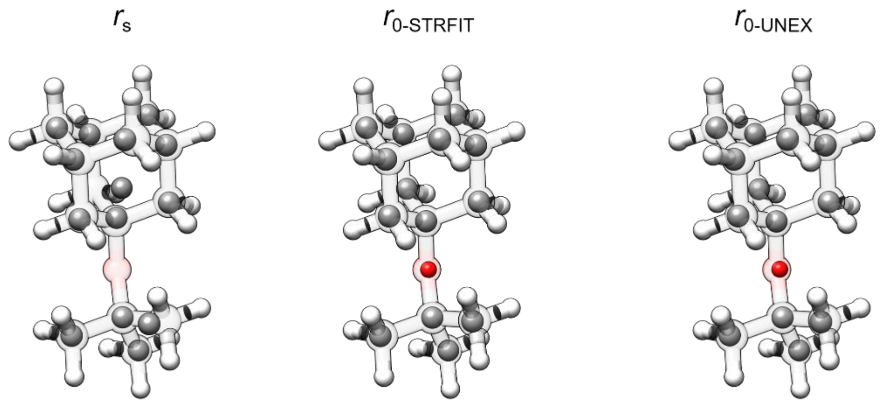

Figure S3. Overlay of the experimental (r_s and r_0) and theoretical (r_e) structures for ATE.

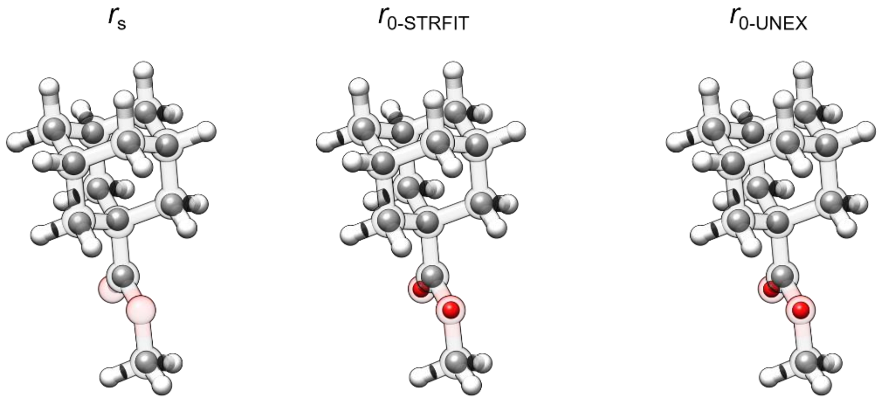

Figure S4. Overlay of the experimental (r_s and r_0) and theoretical (r_e) structures for AMES.

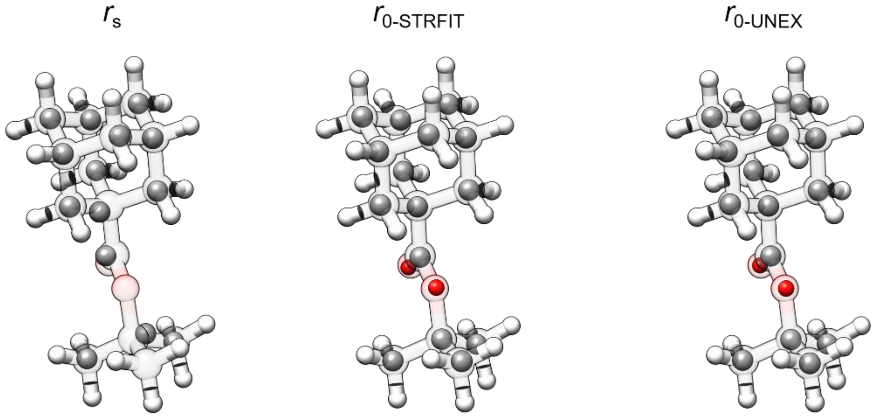

Figure S5. Overlay of the experimental (r_s and r_0) and theoretical (r_e) structures for ATES.

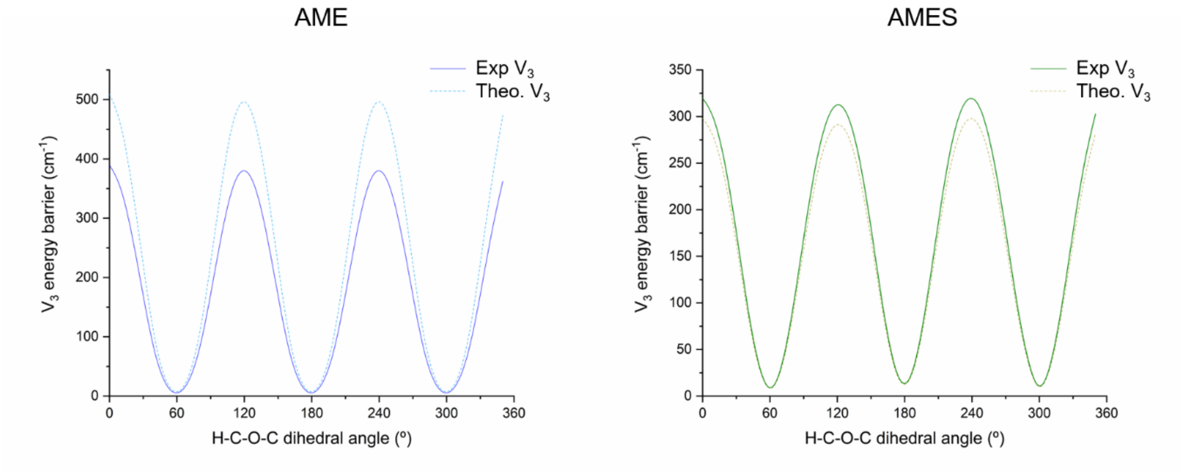

Figure S6. V_3 barrier from the scan of the H–C–O–C coordinate for the internal rotation motion of the methyl top in AME (left) and AMES (right). The dashed lines represent the theoretical V_3 values (B3LYP-D3(BJ)/def2-TZVP) while the solid lines represent an extrapolation of the energy potential considering the experimental V_3 value.

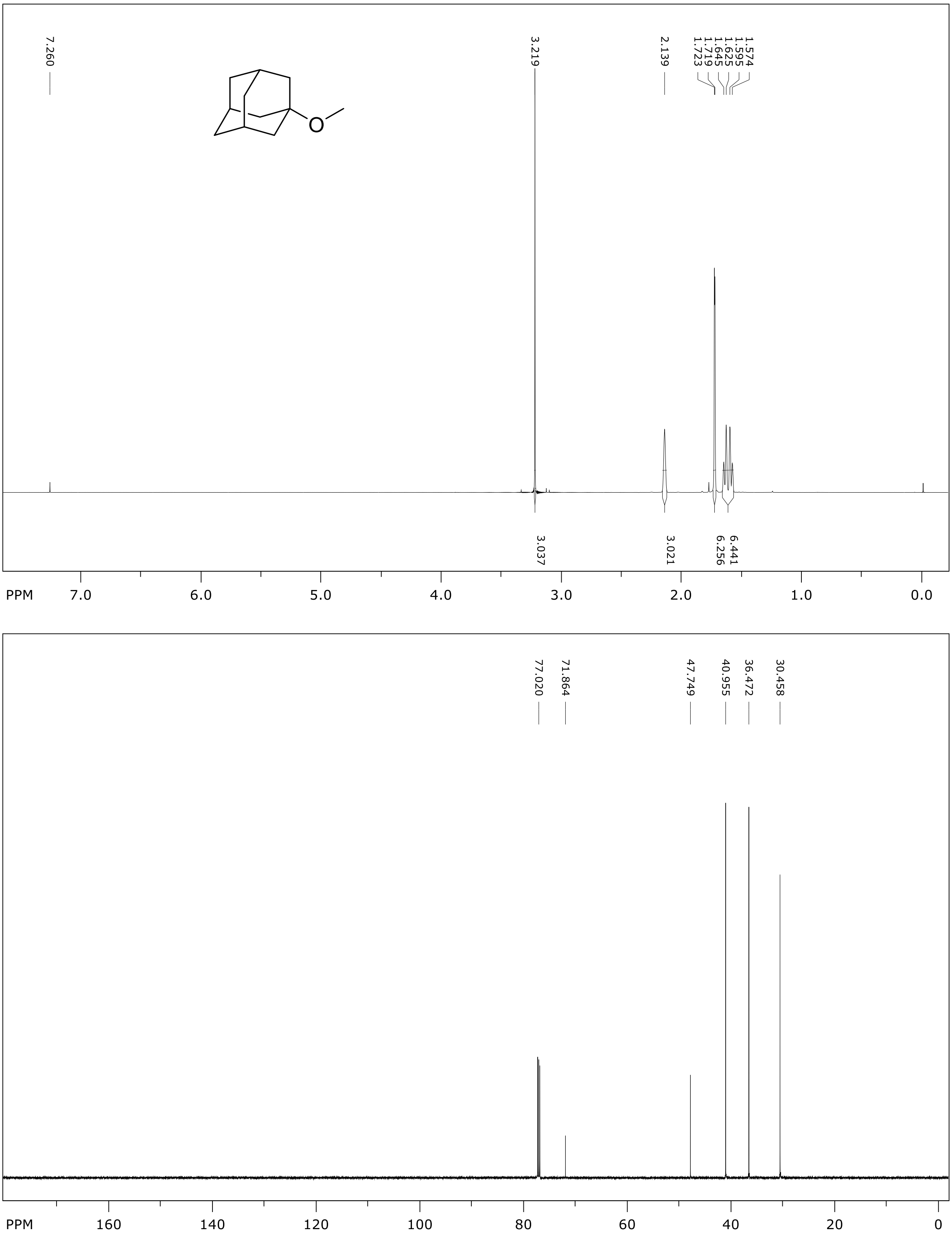

Figure S7. ^{1}H (600 MHz, $CDCl_3$) and ^{13}C NMR (150 MHz, $CDCl_3$) spectra of 1-adamantyl-methyl ether (AME).

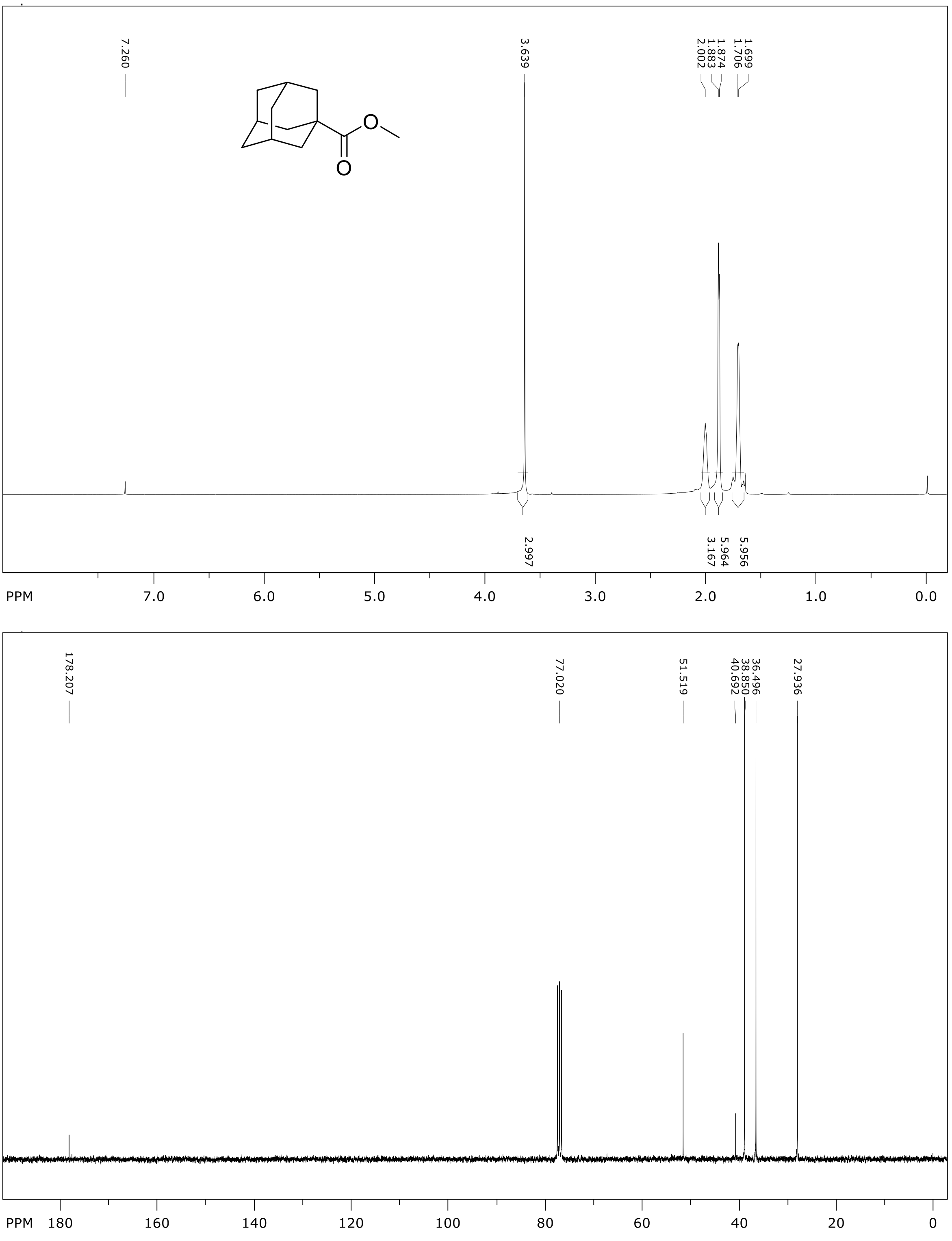

Figure S8. ^{1}H (300 MHz, $CDCl_3$) and ^{13}C NMR (75 MHz, $CDCl_3$) spectra of methyl-1-adamantanecarboxylate (AMES).

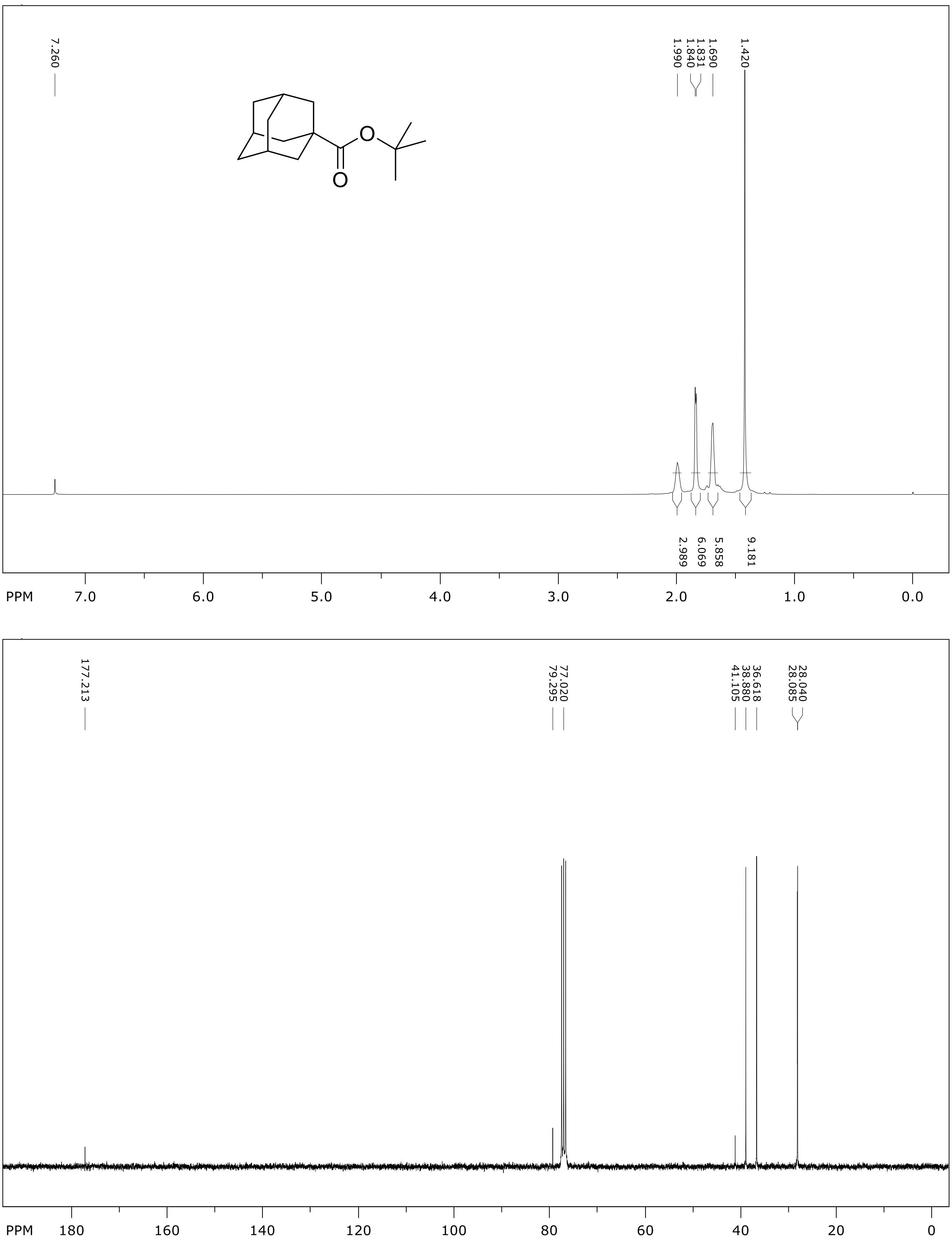

Figure S9. ^{1}H (300 MHz, $CDCl_3$) and ^{13}C NMR (75 MHz, $CDCl_3$) spectra of *tert*-butyl-1-adamantanecarboxylate (ATES).

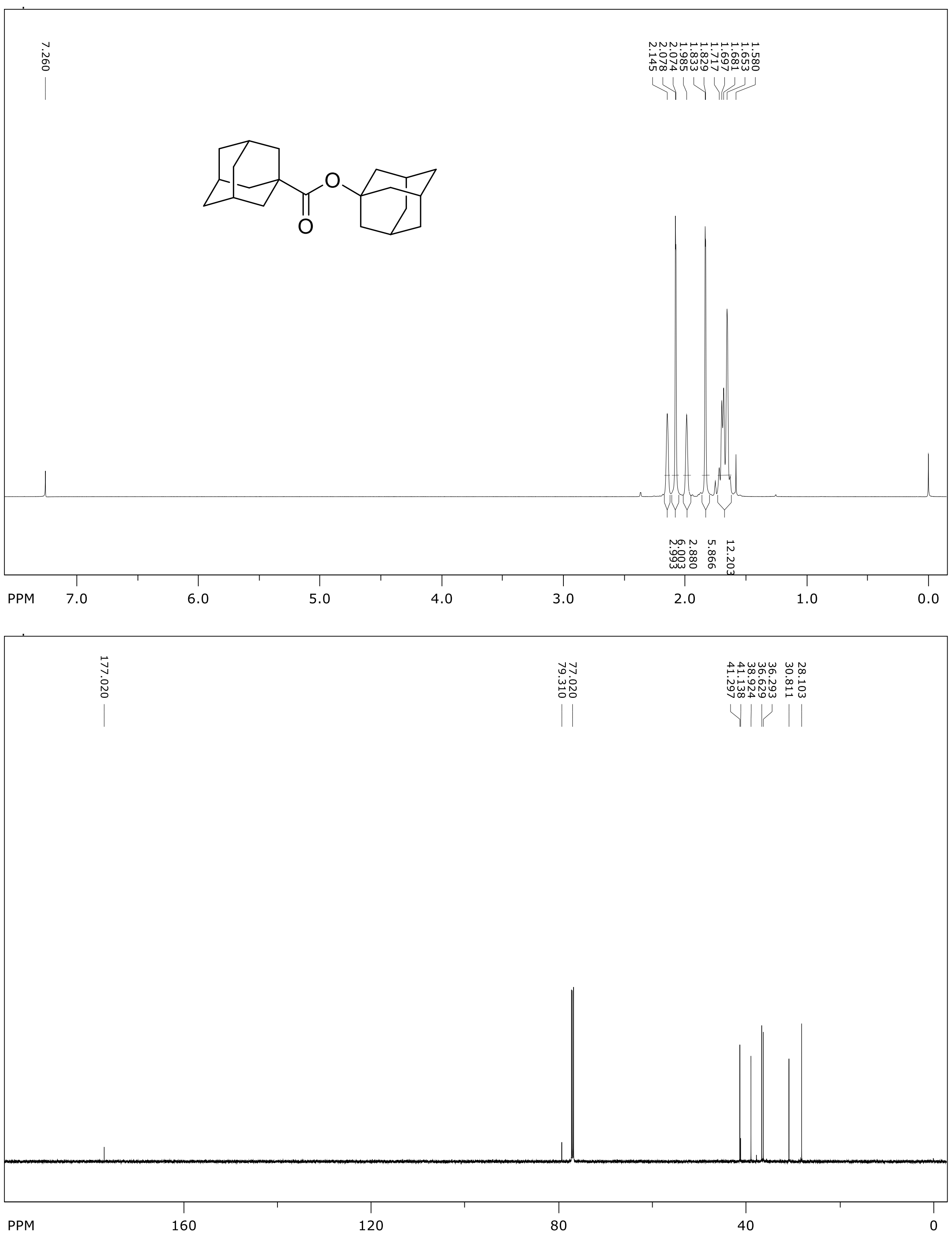

Figure S10. ^{1}H (600 MHz, $CDCl_3$) and ^{13}C NMR (150 MHz, $CDCl_3$) spectra of 1-adamantyl-1-adamantanecarboxylate (DAES).

Table S1. Rotational constants for ATE for the parent and its observed isotopologues fitted with the SPFIT software (see text for details). The centrifugal distortion constants were kept fixed to the parent values.

	Parent	$^{13}C_2$	$^{13}C_3$	$^{13}C_4$	$^{13}C_5$	$^{13}C_6$
A (MHz)[a]	1229.14479(19)	1228.91561(24)	1224.20515(60)	1222.448070(65)	1223.16878(81)	1223.94600(26)
B (MHz)	417.963170(97)	417.95726(17)	417.95311(43)	416.940420(51)	415.80801(60)	416.20749(63)
C (MHz)	417.892195(96)	417.89810(37)	417.3192(13)	416.11458(14)	415.0812(10)	415.68391(45)
$\mu_a/\mu_b/\mu_c$	y/y/Yy	n/y/y	n/y/y	n/y/y	n/y/y	n/y/y
N	241	26	25	23	28	24
σ (kHz)	4.5	2.0	4.2	0.3	9.2	1.8
	$^{13}C_7$	$^{13}C_8$	$^{13}C_9$	$^{13}C_{10}$	$^{13}C_{11}$	$^{13}C_{12}$
A (MHz)	1224.27598(31)	1222.7939(11)	1223.13053(48)	1221.5177(19)	1221.59757(35)	1229.18190(29)
B (MHz)	415.52442(21)	416.70985(84)	417.90534(32)	416.2783(53)	417.7523(11)	415.57039(22)
C (MHz)	414.90224(69)	415.9589(22)	417.16865(96)	415.4618(54)	416.90382(54)	415.49998(73)
$\mu_a/\mu_b/\mu_c$	n/y/y	n/y/y	n/y/y	n/y/y	n/y/y	n/y/y
N	24	26	32	24	24	25
σ (kHz)	1.8	9.1	4.5	13.0	2.1	2.5
	$^{13}C_{13}$	$^{13}C_{14}$	$^{13}C_{15}$			
A (MHz)	1222.0860(12)	1225.818930(49)	1222.4151(15)			
B (MHz)	415.46821(93)	413.204470(57)	414.9779(10)			
C (MHz)	414.7326(24)	412.81832(13)	414.1370(30)			
$\mu_a/\mu_b/\mu_c$	N/Y/Y	N/Y/Y	N/Y/Y			
N	23	23	24			
σ (kHz)	7.4	0.3	10.3			

[a] *A*, *B,* and *C* are the rotational constants. μ_α (α = *a*, ,*b* or *c*) are the electric dipole-moment components, 1 D = 3.33·10^{-30} C·m; y=observed, n=not observed. *N* is the number of fitted transitions. σ is the root-mean square deviation of the fit. [b] Standard error in parentheses in units of the last digit.

Table S2. Rotational constants for AMES for the parent and its observed isotopologues fitted with the SPFIT software for the *A* substate of the internal rotation (see text for details). The centrifugal distortion constants were kept fixed to the parent values.

	Parent	$^{13}C_2$	$^{13}C_4$	$^{13}C_5$=$^{13}C_{11}$	$^{13}C_6$=$^{13}C_{10}$
A (MHz)[a]	1420.37558(35)	1419.79492(87)	1420.2986(13)	1412.5887(10)	1411.4704(10)
B (MHz)	520.87731(19)	519.05741(41)	520.84179(89)	520.04856(44)	518.70735(54)
C (MHz)	494.54309(20)	492.83081(29)	494.49149(45)	494.35871(33)	492.99228(36)
$\mu_a/\mu_b/\mu_c$	y/y/n	y/y/n	y/y/n	y/y/n	y/y/n
N	157	22	12	23	23
σ (kHz)	11.8	8.6	10.6	10.1	10.7
	$^{13}C_7$=$^{13}C_9$	$^{13}C_8$	$^{13}C_{12}$	$^{13}C_{13}$	$^{13}C_{14}$
A (MHz)	1413.07200(64)	1413.47579(89)	1411.0459(12)	1410.14827(78)	1418.7008(18)
B (MHz)	517.24295(26)	518.98103(43)	520.81755(89)	518.74879(34)	512.61696(78)
C (MHz)	491.86850(23)	491.99549(28)	493.35018(46)	491.38103(30)	486.89245(49)
$\mu_a/\mu_b/\mu_c$	y/y/n	y/y/n	y/y/n	y/y/n	y/y/n
N	26	18	14	19	15
σ (kHz)	6.7	7.8	10.6	6.9	12.1

[a] Definitions in Table S1.

Table S3. Rotational constants for ATES for the parent and its observed isotopologues fitted with the SPFIT software (see text for details). The centrifugal distortion constants were kept fixed to the parent values.

	Parent	$^{13}C_2$	$^{13}C_4$	$^{13}C_5$=$^{13}C_{11}$	$^{13}C_6$=$^{13}C_{10}$	$^{13}C_7$=$^{13}C_9$
A (MHz)[a]	1054.68846(23)	1053.6756(16)	1054.52328(47)	1050.59074(90)	1049.39731(70)	1051.00936(59)
B (MHz)	290.628679(71)	[290.46489][b]	290.582(11)	290.272(11)	289.49871(94)	288.72070(89)
C (MHz)	280.036130(73)	279.8327(45)	280.0180(97)	279.888(12)	279.09180(40)	278.48420(42)
$\mu_a/\mu_b/\mu_c$	n/y/n	n/y/n	n/y/n	n/y/n	n/y/n	n/y/n
N	143	4	5	9	14	11
σ (kHz)	7.3	5.7	1.3	6.1	6.0	4.3
	$^{13}C_8$	$^{13}C_{12}$	$^{13}C_{13}$	$^{13}C_{14}$	$^{13}C_{15}$=$^{13}C_{17}$	$^{13}C_{16}$
A (MHz)	1051.67279(92)	1048.02332(66)	1049.3451(11)	1054.5237(17)	1050.83762(45)	[1048.01217]
B (MHz)	289.29786(32)	289.4909(99)	290.3377(93)	288.8660(42)	288.08359(75)	[288.38916]
C (MHz)	278.5790(14)	278.524(13)	279.3731(90)	278.4425(13)	277.88814(36)	[277.49046]
$\mu_a/\mu_b/\mu_c$	n/y/n	n/y/n	n/y/n	n/y/n	n/y/n	
N	7	6	10	6	12	
σ (kHz)	5.3	2.9	8.9	6.7	3.8	

[a] Definitions in Table S1. [b] Parameters in square brackets were fixed to the prediction.

Table S4. Atomic coordinates from the experimental (r_s and r_0) and theoretical (r_e) structures for ATE.

		r_s	$r_{0\text{-STRFIT}}$	$r_{0\text{-UNEX}}$	r_e
C_2	*a*	[0.0][a]	0.10(2)	0.10	0.09
	b	-0.12(1)	-0.14(3)	-0.13	-0.11
	c	0.247(6)	0.27(2)	0.27	0.28
C_3	*a*	-0.12(1)	-0.21(3)	-0.21	-0.21
	b	1.240(7)	1.25(3)	1.24	1.21
	c	-0.35(3)	-0.3(2)	-0.37	-0.44
C_4	*a*	-1.7134(9)	-1.718(6)	-1.72	-1.72
	b	1.398(3)	1.39(7)	1.36	1.32
	c	-0.557(8)	-0.6(2)	-0.65	-0.73
C_5	*a*	-2.4935(7)	-2.497(5)	-2.50	-2.50
	b	1.252(7)	1.2(1)	1.26	1.29
	c	0.68(1)	0.7(1)	0.67	0.59
C_6	*a*	-2.2104(7)	-2.214(5)	-2.21	-2.21
	b	[0.0]	-0.2(2)	-0.11	-0.03
	c	1.343(6)	1.32(5)	1.33	1.32
C_7	*a*	-2.6616(6)	-2.664(4)	-2.66	-2.66
	b	-1.256(4)	-1.25(3)	-1.23	-1.21
	c	0.27(2)	0.3(1)	0.37	0.45
C_8	*a*	-1.886(1)	-1.890(6)	-1.88	-1.88
	b	-1.14(2)	-1.1(2)	-1.13	-1.18
	c	-0.92(2)	-1.0(1)	-0.95	-0.87
C_9	*a*	-0.348(5)	-0.37(3)	-0.37	-0.37
	b	-1.240(6)	-1.2(1)	-1.26	-1.29
	c	-0.70(1)	-0.7(2)	-0.67	-0.59
C_{10}	*a*	-2.167(3)	-2.169(5)	-2.16	-2.16
	b	0.4(2)	0.3(2)	0.24	0.14
	c	-1.56(4)	-1.58(7)	-1.59	-1.61
C_{11}	*a*	-0.684(3)	-0.69(2)	-0.71	-0.71
	b	-0.86(1)	-0.3(2)	-0.24	-0.14
	c	1.34(1)	1.58(7)	1.58	1.59
C_{12}	*a*	2.6418(6)	2.644(4)	2.65	2.65
	b	[0.0]	0.01(2)	0.01	0.01
	c	[0.0]	-0.04(4)	-0.02	-0.02
C_{13}	*a*	2.6531(8)	2.657(5)	2.64	2.64
	b	[0.0]	-0.4(2)	-0.44	-0.53
	c	-1.57(1)	-1.50(4)	-1.49	-1.46
C_{14}	*a*	3.7199(4)	3.722(3)	3.72	3.72
	b	-0.772(4)	-0.79(9)	-0.78	-0.73
	c	0.735(4)	0.7(1)	0.74	0.78
C_{15}	*a*	2.9476(9)	2.950(4)	2.96	2.96
	b	1.52(2)	1.51(5)	1.51	1.51
	c	[0.0]	-0.1(2)	-0.08	-0.02

[a] Imaginary coordinates were kept fixed to zero.

Table S5. Structural parameters from the experimental (r_s and r_0) and theoretical (r_e) structures for ATE.

Parameter	r_s	$r_{0\text{-STRFIT}}$	$r_{0\text{-UNEX}}$	r_e
O_1–C_2	-	1.42(3)	1.440(7)	1.43
O_1–C_{12}	-	1.45(4)	1.441(6)	1.44
C_2–C_3	1.50(2)	1.54(8)	1.538(4)	1.54
C_2–C_9	1.50(1)	1.5(1)	1.546(5)	1.54
C_2–C_{11}	1.49(1)	1.53(7)	1.540(5)	1.54
C_3–C_4	1.61(1)	1.54(6)	1.538(5)	1.54
C_4–C_5	1.47(1)	1.5(2)	1.541(5)	1.53
C_4–C_{10}	1.5(1)	1.5(2)	1.533(5)	1.54
C_5–C_6	1.45(3)	1.5(2)	1.543(5)	1.53
C_6–C_7	1.71(3)	1.5(2)	1.538(5)	1.53
C_6–C_{11}	1.75(2)	1.55(3)	1.534(7)	1.53
C_7–C_8	1.42(2)	1.5(2)	1.539(6)	1.53
C_8–C_9	1.556(6)	1.55(5)	1.540(7)	1.54
C_8–C_{10}	1.7(2)	1.5(2)	1.535(6)	1.54
C_{12}–C_{13}	1.57(2)	1.52(8)	1.537(6)	1.53
C_{12}–C_{14}	1.52(1)	1.54(8)	1.531(4)	1.53
C_{12}–C_{15}	1.55(3)	1.54(6)	1.541(6)	1.53
O_1–C_2–C_3	-	116(3)	115.7(4)	115.5
O_1–C_2–C_9	-	112(4)	111.9(4)	111.9
O_1–C_2–C_{11}	-	103(3)	102.7(4)	103.1
O_1–C_{12}–C_{13}	-	115(3)	114.7(4)	114.4
O_1–C_{12}–C_{14}	-	101(4)	102.2(3)	102.5
O_1–C_{12}–C_{15}	-	111(6)	111.1(4)	111.0
C_2–C_3–C_4	102.8(8)	110(1)	109.8(3)	109.9
C_2–C_9–C_8	105.7(9)	110(8)	110.1(3)	110.3
C_2–C_{11}–C_6	99(1)	110(5)	111.3(2)	111.1
C_2–O_1–C_{12}	-	127(4)	126.5(4)	127.1
C_3–C_4–C_5	114(1)	110(1)	109.7(3)	109.7
C_3–C_2–C_9	114(1)	109(7)	109.7(3)	109.4
C_3–C_2–C_{11}	135(1)	108.3(8)	108.2(4)	108.2
C_4–C_5–C_6	111.6(6)	109.3(5)	109.1(3)	109.3
C_4–C_{10}–C_8	108(3)	110(8)	109.8(2)	109.5
C_5–C_6–C_7	107.2(7)	109(7)	109.3(2)	109.5
C_5–C_4–C_{10}	110(3)	109(6)	109.3(2)	109.4
C_6–C_7–C_8	108.7(8)	109.5(5)	109.4(2)	109.3
C_7–C_6–C_{11}	82(1)	109(2)	109.3(3)	109.4
C_7–C_8–C_9	114(1)	110(10)	109.8(4)	109.8
C_7–C_8–C_{10}	108(2)	109.4(8)	109.3(3)	109.6
C_9–C_8–C_{10}	106(1)	108.7(9)	109.2(4)	109.0
C_{13}–C_{12}–C_{14}	119(2)	109(3)	109.0(4)	109.0
C_{13}–C_{12}–C_{15}	90(7)	111(10)	110.3(4)	110.5
C_{14}–C_{12}–C_{15}	111(4)	108(2)	109.0(3)	109.1

Table S6. Atomic coordinates from the experimental (r_s and r_0) and theoretical (r_e) structures for AMES.

		r_s	$r_{0\text{-STRFIT}}$	$r_{0\text{-UNEX}}$	r_e
C_2	*a*	1.8463(8)	1.851(5)	1.85	1.86
	b	0.385(4)	0.39(2)	0.39	0.40
	c	[0.0][a]	0.00(3)	0.00	0.00
C_4	*a*	0.277(6)	0.35(2)	0.35	0.35
	b	0.173(9)	0.17(4)	0.17	0.18
	c	[0.0]	0.00(8)	0.00	0.00
C_5	*a*	[0.0]	-0.03(5)	-0.06	-0.07
	b	-0.622(4)	-0.62(1)	-0.62	-0.64
	c	1.256(2)	1.261(6)	1.26	1.24
C_6	*a*	-1.584(1)	-1.591(5)	-1.59	-1.59
	b	-0.827(2)	-0.828(8)	-0.83	-0.85
	c	1.255(1)	1.258(6)	1.26	1.24
C_7	*a*	-2.294(1)	-2.298(4)	-2.30	-2.29
	b	0.532(4)	0.53(1)	0.53	0.51
	c	1.254(2)	1.259(6)	1.26	1.27
C_8	*a*	-1.8832(8)	-1.887(4)	-1.89	-1.89
	b	1.325(1)	1.327(6)	1.32	1.32
	c	[0.0]	0.01(1)	0.01	0.03
C_9	*a*	-2.294(1)	-2.297(4)	-2.30	-2.30
	b	0.532(4)	0.53(1)	0.53	0.56
	c	-1.254(2)	-1.257(6)	-1.26	-1.24
C_{10}	*a*	-1.584(1)	-1.590(5)	-1.59	-1.59
	b	-0.827(2)	-0.828(9)	-0.82	-0.80
	c	-1.255(1)	-1.260(6)	-1.26	-1.27
C_{11}	*a*	[0.0]	-0.07(4)	-0.06	-0.07
	b	-0.622(4)	-0.62(1)	-0.61	-0.59
	c	-1.256(2)	-1.261(5)	-1.26	-1.27
C_{12}	*a*	-1.9947(8)	-1.997(4)	-2.00	-1.99
	b	-1.6155(9)	-1.618(5)	-1.61	-1.62
	c	[0.0]	-0.01(1)	-0.01	-0.03
C_{13}	*a*	-0.339(5)	-0.36(2)	-0.37	-0.37
	b	1.537(1)	1.538(5)	1.54	1.54
	c	[0.0]	0.03(3)	0.01	0.03
C_{14}	*a*	3.9565(4)	3.959(2)	3.96	3.96
	b	-0.658(2)	-0.66(1)	-0.67	-0.67
	c	[0.0]	0.01(4)	0.00	0.00

[a] Imaginary coordinates were kept fixed to zero.

Table S7. Structural parameters from the experimental (r_s and r_0) and theoretical (r_e) structures for AMES.

Parameter	r_s	$r_{0\text{-STRFIT}}$	$r_{0\text{-UNEX}}$	r_e
O_1–C_2	-	1.35(3)	1.354(7)	1.35
O_1–C_{14}	-	1.43(6)	1.428(7)	1.43
C_2–O_3	-	1.21(3)	1.221(7)	1.21
C_2–C_4	1.583(6)	1.52(2)	1.513(7)	1.52
C_4–C_5	1.51(1)	1.54(7)	1.546(7)	1.55
C_4–C_{11}	1.51(1)	1.54(9)	1.546(7)	1.55
C_4–C_{13}	1.496(9)	1.54(4)	1.540(7)	1.54
C_5–C_6	1.60(2)	1.57(5)	1.537(8)	1.53
C_6–C_7	1.533(4)	1.53(1)	1.528(6)	1.53
C_6–C_{12}	1.54(1)	1.55(1)	1.551(6)	1.54
C_7–C_8	1.54(2)	1.54(1)	1.535(6)	1.53
C_8–C_9	1.54(2)	1.55(1)	1.553(6)	1.53
C_8–C_{13}	1.559(5)	1.54(2)	1.535(7)	1.53
C_9–C_{10}	1.533(4)	1.53(1)	1.528(6)	1.53
C_{10}–C_{11}	1.60(2)	1.53(4)	1.537(8)	1.53
C_{10}–C_{12}	1.54(1)	1.53(1)	1.535(7)	1.54
O_1–C_2–O_3	-	122(1)	122.1(4)	122.6
O_1–C_2–C_4	-	112(2)	112.1(4)	111.7
C_2–O_1–C_{14}	-	115(4)	115.9(5)	115.9
C_2–C_4–C_5	105(1)	109(4)	110.0(6)	110.2
C_2–C_4–C_{11}	105(1)	110(3)	109.8(6)	109.9
C_2–C_4–C_{13}	106.6(5)	109(2)	109.5(5)	109.8
O_3–C_2–C_4	-	126(3)	125.8(5)	125.6
C_4–C_5–C_6	104.4(7)	108(2)	109.6(4)	109.8
C_4–C_{13}–C_8	106.5(3)	110(1)	109.8(3)	110.0
C_4–C_{11}–C_{10}	104.4(7)	110(2)	109.6(4)	109.8
C_5–C_6–C_7	110.2(2)	109.7(7)	109.9(4)	109.7
C_5–C_6–C_{12}	109.3(2)	109.3(5)	109.4(4)	109.4
C_6–C_7–C_8	109.4(3)	109.6(5)	109.6(1)	109.4
C_6–C_{12}–C_{10}	109.3(1)	109.6(3)	109.5(2)	109.6
C_7–C_6–C_{12}	109.3(2)	109.4(4)	109.1(2)	109.5
C_7–C_8–C_9	109.1(2)	109.3(6)	109.1(2)	109.5
C_7–C_8–C_{13}	109(1)	109(1)	109.7(4)	109.6
C_8–C_9–C_{10}	109.4(3)	109.5(3)	109.4(1)	109.5
C_9–C_8–C_{13}	109(1)	110(1)	109.4(4)	109.7
C_9–C_{10}–C_{11}	110.2(2)	109.6(7)	109.9(4)	109.5
C_9–C_{10}–C_{12}	109.3(2)	109.4(5)	109.4(2)	109.5
C_{11}–C_4–C_{13}	114(1)	110(4)	109.4(5)	109.1
C_{11}–C_{10}–C_{12}	109.3(2)	109.4(4)	109.5(4)	109.4

Table S8. Atomic coordinates from the experimental (r_s and r_0) and theoretical (r_e) structures for ATES.

		r_s	$r_{0\text{-STRFIT}}$	$r_{0\text{-UNEX}}$	r_e
C_2	*a*	0.957(8)	1.02(2)	0.99	0.99
	b	0.63(1)	0.68(2)	0.68	0.70
	c	-0.25(3)	-0.02(1)	0.00	-0.01
C_4	*a*	-0.40(6)	-0.46(2)	-0.48	-0.48
	b	[0.0][a]	0.31(2)	0.29	0.31
	c	-0.35(7)	-0.02(1)	-0.01	-0.01
C_5	*a*	-0.78(3)	-0.79(2)	-0.81	-0.81
	b	-0.58(4)	-0.50(3)	-0.48	-0.47
	c	1.24(2)	1.29(2)	1.28	1.29
C_6	*a*	-2.2877(9)	-2.295(8)	-2.30	-2.30
	b	-0.923(2)	-0.93(2)	-0.87	-0.86
	c	1.256(2)	1.27(2)	1.29	1.30
C_7	*a*	-3.1515(7)	-3.168(6)	-3.15	-3.16
	b	0.346(6)	0.36(3)	0.40	0.41
	c	1.254(2)	1.24(2)	1.24	1.22
C_8	*a*	-2.834(1)	-2.842(7)	-2.84	-2.84
	b	1.191(2)	1.18(2)	1.17	1.17
	c	[0.0]	-0.06(1)	-0.07	-0.07
C_9	*a*	-3.1515(7)	-3.157(7)	-3.15	-3.15
	b	0.346(6)	0.36(3)	0.28	0.27
	c	-1.254(2)	-1.26(1)	-1.27	-1.28
C_{10}	*a*	-2.2877(9)	-2.292(8)	-2.28	-2.28
	b	-0.923(2)	-0.94(2)	-0.97	-0.99
	c	-1.256(2)	-1.24(1)	-1.22	-1.21
C_{11}	*a*	-0.78(3)	-0.81(2)	-0.80	-0.80
	b	-0.58(4)	-0.57(2)	-0.59	-0.60
	c	-1.24(2)	-1.25(1)	-1.24	-1.22
C_{12}	*a*	-2.60(1)	-2.614(9)	-2.60	-2.60
	b	-1.74(1)	-1.76(1)	-1.75	-1.75
	c	0.2(1)	0.05(2)	0.07	0.09
C_{13}	*a*	-1.34(1)	-1.35(2)	-1.36	-1.36
	b	1.58(1)	1.57(2)	1.56	1.56
	c	[0.0]	0.06(2)	-0.08	-0.09
C_{14}	*a*	3.231(2)	3.236(7)	3.24	3.24
	b	[0.0]	-0.31(5)	-0.29	-0.30
	c	0.42(2)	0.019(6)	0.00	0.00
C_{15}	*a*	3.7111(5)	3.726(7)	3.72	3.74
	b	0.412(5)	0.45(5)	0.44	0.42
	c	-1.267(2)	-1.24(2)	-1.26	-1.25
C_{16}	*a*	-[b]	3.63(4)	3.67	3.67
	b	-	-1.7(1)	-1.76	-1.76
	c	-	-0.09(2)	-0.02	-0.01
C_{17}	*a*	3.7111(5)	3.720(7)	3.71	3.71
	b	0.412(5)	0.33(6)	0.37	0.39
	c	1.267(2)	1.29(2)	1.28	1.28

[a] Imaginary coordinates were kept fixed to zero. [b] No lines found for C_{16}.

Table S9. Structural parameters from the experimental (r_s and r_0) and theoretical (r_e) structures for ATES.

Parameter	r_s	$r_{0\text{-STRFIT}}$	$r_{0\text{-UNEX}}$	r_e
$O_1–C_2$	-	1.29(7)	1.33(1)	1.34
$O_1–C_{14}$	-	1.48(4)	1.47(1)	1.47
$C_2–O_3$	-	1.2(1)	1.230(7)	1.21
$C_2–C_4$	1.50(7)	1.52(3)	1.52(1)	1.52
$C_4–C_5$	1.73(7)	1.54(2)	1.543(9)	1.55
$C_4–C_{11}$	1.13(8)	1.59(2)	1.543(9)	1.55
$C_4–C_{13}$	1.9(1)	1.55(3)	1.547(9)	1.54
$C_5–C_6$	1.54(3)	1.56(2)	1.54(1)	1.53
$C_6–C_7$	1.535(5)	1.55(3)	1.53(1)	1.53
$C_6–C_{12}$	1.4(1)	1.51(2)	1.53(1)	1.53
$C_7–C_8$	1.54(1)	1.58(2)	1.555(9)	1.53
$C_8–C_9$	1.54(1)	1.48(2)	1.533(9)	1.53
$C_8–C_{13}$	1.54(1)	1.54(3)	1.53(1)	1.53
$C_9–C_{10}$	1.535(5)	1.56(2)	1.53(1)	1.53
$C_{10}–C_{11}$	1.54(3)	1.53(2)	1.53(1)	1.53
$C_{10}–C_{12}$	1.7(1)	1.56(1)	1.54(1)	1.53
$C_{14}–C_{15}$	1.80(2)	1.51(2)	1.534(8)	1.53
$C_{14}–C_{16}$	-	1.5(2)	1.53(1)	1.52
$C_{14}–C_{17}$	1.05(1)	1.53(2)	1.516(9)	1.52
$O_1–C_2–O_3$	-	[124.6]	124.0(8)	124.6
$O_1–C_2–C_4$	-	[110.8]	111.7(5)	110.8
$O_1–C_{14}–O_{15}$	-	[110.0]	109.1(6)	110.0
$O_1–C_{14}–O_{16}$	-	[102.7]	102.7(7)	102.7
$O_1–C_{14}–O_{17}$	-	[110.0]	110.5(7)	110.0
$C_2–O_1–C_{14}$	-	[122.2]	122.8(7)	122.2
$C_2–C_4–C_5$	107(4)	[109.6]	109.3(7)	109.6
$C_2–C_4–C_{11}$	124(5)	[110.5]	110.5(7)	110.5
$C_2–C_4–C_{13}$	95(5)	[110.0]	109.9(5)	110.0
$O_3–C_2–C_4$	-	[124.6]	124.3(7)	124.6
$C_4–C_5–C_6$	107(2)	[110.0]	109.8(5)	110.0
$C_4–C_{13}–C_8$	106(2)	[110.1]	109.9(4)	110.1
$C_4–C_{11}–C_{10}$	117(4)	[110.0]	109.6(5)	110.0
$C_5–C_6–C_7$	111(2)	[109.6]	109.1(6)	109.6
$C_5–C_6–C_{12}$	110(2)	[109.4]	109.4(6)	109.4
$C_6–C_7–C_8$	109.7(2)	[109.4]	109.2(4)	109.4
$C_6–C_{12}–C_{10}$	109(2)	[109.6]	109.7(3)	109.6
$C_7–C_6–C_{12}$	111(2)	[109.5]	109.6(4)	109.5
$C_7–C_8–C_9$	108.5(3)	[109.5]	109.0(4)	109.5
$C_7–C_8–C_{13}$	110(3)	[109.5]	109.2(6)	109.5
$C_8–C_9–C_{10}$	109.7(2)	[109.4]	109.4(4)	109.4
$C_9–C_8–C_{13}$	110(3)	[109.5]	110.2(6)	109.5
$C_9–C_{10}–C_{11}$	111(2)	[109.7]	110.4(6)	109.7
$C_9–C_{10}–C_{12}$	107(1)	[109.4]	109.1(4)	109.4
$C_{11}–C_4–C_{13}$	114(6)	[109.1]	108.6(7)	109.1
$C_{11}–C_{10}–C_{12}$	106(1)	[109.4]	109.5(6)	109.4
$C_{15}–C_{14}–C_{16}$	-	[110.7]	110.9(7)	110.7
$C_{15}–C_{14}–C_{17}$	123(1)	[112.4]	112.9(7)	112.4
$C_{16}–C_{14}–C_{17}$	-	[110.7]	110.3(7)	110.7

Table S10. Comparison of experimental (r_s and r_0) and theoretical (r_e) selected bond distances.

	O_1–C_2			O_1–$C_{12/14}$		
	$r_{0\text{-STRFIT}}$	$r_{0\text{-UNEX}}$	r_e	$r_{0\text{-STRFIT}}$	$r_{0\text{-UNEX}}$	r_e
AME	-	-	1.43	-	-	1.41
ATE	1.42(3)	1.440(7)	1.43	1.45(4)	1.441(6)	1.44
DAE	1.42(1)	1.4(3)	1.43	1.42(1)	1.4(3)	1.43
AMES	1.34(3)	1.355(7)	1.35	1.43(6)	1.428(7)	1.43
ATES	1.29(7)	1.33(1)	1.34	1.48(4)	1.47(1)	1.47
DAES	-	-	1.34	-	-	1.46

Table S11. Observed frequencies and residuals (MHz) for AME for $J'K_a'K_c' \leftarrow J''K_a''K_c''$ transitions using the XIAM program.

J'	K_a'	K_c'	J''	K_a''	K_c''	S	Obs	Res
2	1	2	1	1	1	A	3350.0531	0.0082
2	1	2	1	1	1	E	3350.1054	0.0016
2	0	2	1	0	1	A	3360.9006	-0.0031
2	0	2	1	0	1	E	3360.9006	0.0178
2	1	1	1	1	0	A	3371.9916	-0.0003
2	1	1	1	1	0	E	3371.9198	-0.0003
3	1	3	2	1	2	A	5025.0049	0.0091
3	1	3	2	1	2	E	5025.0049	0.0001
3	0	3	2	0	2	A	5041.0624	-0.0056
3	0	3	2	0	2	E	5041.0624	0.0263
3	2	2	2	2	1	A	5041.5449	0.0192
3	2	2	2	2	1	E	5041.7102	-0.0128
3	2	1	2	2	0	A	5042.0015	0.0155
3	2	1	2	2	0	E	5041.7790	0.0183
3	1	2	2	1	1	A	5057.9156	0.0001
3	1	2	2	1	1	E	5057.8835	-0.0042
4	1	4	3	1	3	A	6699.8628	0.0009
4	1	4	3	1	3	E	6699.8628	0.0027
4	0	4	3	0	3	A	6720.8811	-0.0065
4	0	4	3	0	3	E	6720.8811	0.0371
4	2	3	3	2	2	A	6721.9436	-0.0012
4	2	3	3	2	2	E	6722.3499	0.0011
4	3	2	3	3	1	A	6722.2506	-0.0074
4	3	2	3	3	1	E	6722.2506	0.0096
4	3	1	3	3	0	A	6722.2506	-0.0119
4	3	1	3	3	0	E	6722.2506	0.0101
4	2	2	3	2	1	A	6723.0940	-0.0008
4	2	2	3	2	1	E	6722.6555	0.0019
4	1	3	3	1	2	A	6743.7602	0.0088
4	1	3	3	1	2	E	6743.7118	-0.0170
1	1	1	0	0	0	A	2459.7355	-0.0052
1	1	1	0	0	0	E	2459.6176	-0.0133
2	0	2	1	1	1	A	2581.6756	0.0033
2	0	2	1	1	1	E	2581.7213	-0.0296
4	2	2	3	3	1	A	2799.9999	-0.0134
2	1	2	1	0	1	A	4129.2665	-0.0098
2	1	2	1	0	1	E	4129.2350	-0.0008
3	0	3	2	1	2	A	4272.7357	0.0403
3	0	3	2	1	2	E	4272.6892	0.0061
4	1	4	3	2	1	A	4312.1599	0.0165
4	1	4	3	2	1	E	4310.4017	0.0302
4	1	3	3	2	2	A	4422.4633	0.0150
5	2	4	4	3	1	A	4478.2941	-0.0176
5	2	3	4	3	2	A	4482.3555	0.0152
2	2	1	1	1	0	A	5709.6774	-0.0074
2	2	1	1	1	0	E	5707.5506	0.0185
2	2	0	1	1	1	A	5720.7700	-0.0033
2	2	0	1	1	1	E	5722.8126	-0.0239
3	1	3	2	0	2	A	5793.3759	0.0075
3	1	3	2	0	2	E	5793.3302	-0.0275
5	1	5	4	2	2	A	5963.6641	-0.0012
5	1	5	4	2	2	E	5962.3226	-0.0072
4	0	4	3	1	3	A	5968.5901	0.0029
4	0	4	3	1	3	E	5968.5901	0.0678
5	1	4	4	2	3	A	6129.9708	-0.0009
5	1	4	4	2	3	E	6131.3509	-0.0258
6	2	5	5	3	2	A	6157.8937	-0.0105
6	2	4	5	3	3	A	6165.9612	-0.0013
3	2	2	2	1	1	A	7379.2172	-0.0014
3	2	2	2	1	1	E	7377.3286	-0.0063
3	2	1	2	1	2	A	7412.7189	0.0046
3	2	1	2	1	2	E	7414.4948	0.0014
4	1	4	3	0	3	A	7452.1456	-0.0167
4	1	4	3	0	3	E	7452.1456	-0.0361
6	1	6	5	2	3	A	7608.3098	-0.0055
6	1	6	5	2	3	E	7607.4869	-0.0170
5	0	5	4	1	4	A	7668.9634	-0.0110
7	2	6	6	3	3	A	7836.8718	0.0019
6	1	5	5	2	4	A	7842.7179	-0.0004
6	1	5	5	2	4	E	7843.5911	-0.0220
8	2	6	8	1	7	A	2183.1590	-0.0025
8	2	6	8	1	7	E	2183.3347	0.0430
7	2	5	7	1	6	A	2216.5048	-0.0027
7	2	5	7	1	6	E	2216.7903	0.0422
6	2	4	6	1	5	A	2247.8475	-0.0050
6	2	4	6	1	5	E	2248.3337	0.0314
5	2	3	5	1	4	A	2276.3350	-0.0037
5	2	3	5	1	4	E	2277.1882	0.0273
4	2	2	4	1	3	A	2301.2208	-0.0011
4	2	2	4	1	3	E	2302.5668	0.0185
3	2	1	3	1	2	A	2321.8698	-0.0086
3	2	1	3	1	2	E	2323.6270	0.0035
2	2	0	2	1	1	A	2337.8017	-0.0062
2	2	1	2	1	2	A	2370.6059	-0.0074
3	2	2	3	1	3	A	2387.1549	0.0117
3	2	2	3	1	3	E	2385.3152	0.0029
4	2	3	4	1	4	A	2409.2253	-0.0007
4	2	3	4	1	4	E	2407.8017	0.0007
5	2	4	5	1	5	A	2436.8933	-0.0031
5	2	4	5	1	5	E	2435.9631	0.0050

Table S11. Continued.

J'	K_a'	K_c'	J''	K_a''	K_c''	S	Obs	Res
6	2	5	6	1	6	A	2470.1877	-0.0072
6	2	5	6	1	6	E	2469.6112	0.0053
7	2	6	7	1	7	A	2509.1551	-0.0111
7	2	6	7	1	7	E	2508.7866	0.0307
8	2	7	8	1	8	A	2553.8410	-0.0160
8	2	7	8	1	8	E	2553.5734	0.0566
9	2	8	9	1	9	A	2604.3059	-0.0077
11	3	8	11	2	9	A	3867.8719	-0.0198
10	3	7	10	2	8	A	3884.4258	-0.0172
9	3	6	9	2	7	A	3897.1314	-0.0165
8	3	5	8	2	6	A	3906.5480	-0.0124
8	3	5	8	2	6	E	3909.2546	0.0050
7	3	4	7	2	5	A	3913.2400	-0.0070
7	3	4	7	2	5	E	3915.9291	0.0121
6	3	3	6	2	4	A	3917.7516	-0.0063
6	3	3	6	2	4	E	3920.2608	-0.0058
5	3	2	5	2	3	A	3920.5983	-0.0078
5	3	2	5	2	3	E	3922.7582	-0.0067
4	3	1	4	2	2	A	3922.2424	-0.0075
4	3	1	4	2	2	E	3923.9078	-0.0051
3	3	0	3	2	1	A	3923.0903	0.0080
3	3	0	3	2	1	E	3924.3294	0.0034
5	3	3	5	2	4	A	3924.5977	-0.0106
5	3	3	5	2	4	E	3922.3236	-0.0067
6	3	4	6	2	5	A	3925.7216	-0.0102
6	3	4	6	2	5	E	3923.0903	-0.0111
7	3	5	7	2	6	A	3927.5207	-0.0083
7	3	5	7	2	6	E	3924.7189	-0.0148
9	3	7	9	2	8	A	3934.0128	-0.0111
9	3	7	9	2	8	E	3931.2888	-0.0113
10	3	8	10	2	9	A	3939.2048	-0.0122
7	4	3	7	3	4	A	5491.9247	-0.0049
7	4	3	7	3	4	E	5492.9499	-0.0103
7	4	4	7	3	5	A	5492.0773	-0.0101
7	4	4	7	3	5	E	5490.8832	-0.0118
6	4	2	6	3	3	A	5492.3670	-0.0044
6	4	2	6	3	3	E	5493.3465	-0.0094
6	4	3	6	3	4	A	5492.4275	-0.0071
6	4	3	6	3	4	E	5491.2797	-0.0096
5	4	2	5	3	3	A	5492.6474	-0.0168
5	4	2	5	3	3	E	5491.5306	-0.0097
5	4	1	5	3	2	A	5492.6474	0.0043
5	4	1	5	3	2	E	5493.6001	-0.0070
4	4	1	4	3	2	A	5492.7888	-0.0148
4	4	1	4	3	2	E	5491.6721	-0.0158
4	4	0	4	3	1	A	5492.7888	-0.0095
4	4	0	4	3	1	E	5493.7468	-0.0079
10	5	5	10	4	6	A	7060.7750	0.0014
10	5	6	10	4	7	A	7060.7750	-0.0109
9	5	5	9	4	6	A	7061.2890	0.0077
9	5	4	9	4	5	A	7061.2890	0.0130
8	5	4	8	4	5	A	7061.6572	0.0106
8	5	3	8	4	4	A	7061.6572	0.0127
7	5	3	7	4	4	A	7061.9258	0.0191
7	5	2	7	4	3	A	7061.9258	0.0198
6	5	2	6	4	3	A	7062.1053	0.0211
6	5	1	6	4	2	A	7062.1053	0.0213
5	5	0	5	4	1	A	7062.2180	0.0189
5	5	1	5	4	2	A	7062.2180	0.0189

Table S12. Observed frequencies and residuals (MHz) for the parent species of ATE for $J'K'_aK'_c \leftarrow J''K''_aK''_c$ transitions using the SPFIT program.

J'	K'_a	K'_c	J''	K''_a	K''_c	Obs	Res
3	1	3	2	1	2	2507.4599	0.0006
3	0	3	2	0	2	2507.5660	0.0003
3	2	2	2	2	1	2507.5660	0.0003
3	2	1	2	2	0	2507.5660	0.0003
3	1	2	2	1	1	2507.6725	0.0002
4	1	4	3	1	3	3343.2861	0.0075
4	0	4	3	0	3	3343.4249	0.0043
4	2	3	3	2	2	3343.4249	0.0043
4	3	2	3	3	1	3343.4249	0.0043
4	3	1	3	3	0	3343.4249	0.0043
4	2	2	3	2	1	3343.4249	0.0043
4	1	3	3	1	2	3343.5642	0.0016
5	1	5	4	1	4	4179.0965	-0.0010
5	0	5	4	0	4	4179.2748	-0.0002
5	2	4	4	2	3	4179.2748	-0.0002
5	3	3	4	3	2	4179.2748	-0.0002
5	3	2	4	3	1	4179.2748	-0.0002
5	2	3	4	2	2	4179.2748	-0.0002
5	1	4	4	1	3	4179.4528	0.0003
6	1	6	5	1	5	5014.9174	0.0012
6	0	6	5	0	5	5015.1294	0.0003
6	2	5	5	2	4	5015.1294	0.0003
6	3	4	5	3	3	5015.1294	0.0003
6	3	3	5	3	2	5015.1294	0.0003
6	4	3	5	4	2	5015.1294	0.0003
6	4	2	5	4	1	5015.1294	0.0003
6	2	4	5	2	3	5015.1294	0.0003
6	1	5	5	1	4	5015.3418	-0.0002
7	1	7	6	1	6	5850.7325	-0.0017
7	0	7	6	0	6	5850.9798	-0.0029
7	2	6	6	2	5	5850.9798	-0.0029
7	3	5	6	3	4	5850.9798	-0.0029
7	3	4	6	3	3	5850.9798	-0.0029
7	4	4	6	4	3	5850.9798	-0.0029
7	4	3	6	4	2	5850.9798	-0.0029
7	2	5	6	2	4	5850.9798	-0.0029
7	1	6	6	1	5	5851.2298	-0.0012
8	1	8	7	1	7	6686.5548	0.0030
8	0	8	7	0	7	6686.8389	0.0032
8	2	7	7	2	6	6686.8389	0.0032
8	3	6	7	3	5	6686.8389	0.0032
8	3	5	7	3	4	6686.8389	0.0032
8	4	5	7	4	4	6686.8389	0.0032
8	4	4	7	4	3	6686.8389	0.0032
8	2	6	7	2	5	6686.8389	0.0032
8	1	7	7	1	6	6687.1231	0.0036
9	1	9	8	1	8	7522.3670	-0.0014
9	0	9	8	0	8	7522.6865	-0.0015
9	2	8	8	2	7	7522.6865	-0.0015
9	3	7	8	3	6	7522.6865	-0.0015
9	3	6	8	3	5	7522.6865	-0.0015
9	4	6	8	4	5	7522.6865	-0.0015
9	4	5	8	4	4	7522.6865	-0.0015
9	5	5	8	5	4	7522.6865	-0.0015
9	5	4	8	5	3	7522.6865	-0.0015
9	2	7	8	2	6	7522.6865	-0.0015
9	1	8	8	1	7	7523.0069	-0.0003
4	0	4	3	1	3	2532.4133	-0.0030
2	1	2	1	0	1	2482.8245	0.0032
3	1	3	2	0	2	3318.5733	0.0033
5	0	5	4	1	4	3368.4109	-0.0018
6	0	6	5	1	5	4204.4399	-0.0041
4	1	4	3	0	3	4154.2840	0.0011
5	1	5	4	0	4	4989.9599	0.0000
7	0	7	6	1	6	5040.5125	0.0021
6	1	6	5	0	5	5825.6034	0.0023
8	0	8	7	1	7	5876.6090	-0.0024
7	1	7	6	0	6	6661.2051	-0.0013
9	0	9	8	1	8	6712.7481	0.0009
10	0	10	9	1	9	7548.9229	0.0054
8	1	8	7	0	7	7496.7754	-0.0002
2	1	1	1	0	1	2483.0374	0.0031
4	0	4	3	1	2	2531.9887	-0.0018
6	1	6	5	2	4	2580.7409	0.0083
6	1	5	5	2	3	2582.2103	-0.0125
8	2	6	7	3	4	2630.7503	-0.0002
8	2	7	7	3	5	2630.7503	-0.0002
3	1	2	2	0	2	3318.9991	0.0033
5	0	5	4	1	3	3367.7009	-0.0020
7	1	7	6	2	5	3416.3336	-0.0040
7	1	6	6	2	4	3418.3250	0.0003
9	2	8	8	3	6	3466.6040	0.0012
9	2	7	8	3	5	3466.6040	0.0012
11	3	9	10	4	7	3515.8805	0.0097
11	3	8	10	4	6	3515.8805	0.0097
4	1	3	3	0	3	4154.9933	0.0007
6	0	6	5	1	4	4203.3788	-0.0006
8	1	8	7	2	6	4251.9111	0.0044
8	1	7	7	2	5	4254.4567	-0.0045
10	2	9	9	3	7	4302.4532	-0.0015

Table S12. Continued.

J'	K_a'	K_c'	J''	K_a''	K_c''	Obs	Res
10	2	8	9	3	6	4302.4532	-0.0015
5	1	4	4	0	4	4991.0233	-0.0012
7	0	7	6	1	5	5039.0173	-0.0025
9	1	8	8	2	6	5090.6291	-0.0032
6	1	5	5	0	5	5827.0928	0.0011
8	0	8	7	1	6	5874.6210	-0.0031
10	1	10	9	2	8	5922.9401	0.0040
12	2	10	11	3	8	5974.1569	0.0012
12	2	11	11	3	9	5974.1569	0.0012
7	1	6	6	0	6	6663.1939	0.0002
9	0	9	8	1	7	6710.1806	-0.0114
8	1	7	7	0	7	7499.3304	-0.0003
10	0	10	9	1	8	7545.7147	-0.0088
2	2	0	1	1	0	4105.3202	-0.0062
2	2	0	1	1	1	4105.3202	-0.0062
2	2	1	1	1	0	4105.3993	0.0019
2	2	1	1	1	1	4105.3993	0.0019
3	2	1	2	1	2	4941.3337	0.0102
3	2	2	2	1	2	4941.3337	0.0102
4	2	3	3	1	2	5776.8637	0.0046
4	2	2	3	1	2	5776.8637	0.0046
4	2	3	3	1	3	5777.2854	0.0006
4	2	2	3	1	3	5777.2854	0.0006
3	3	0	2	2	0	6563.6522	0.0010
3	3	0	2	2	1	6563.6522	0.0010
3	3	1	2	2	0	6563.6522	0.0010
3	3	1	2	2	1	6563.6522	0.0010
5	2	4	4	1	3	6612.5759	0.0042
5	2	3	4	1	3	6612.5759	0.0042
5	2	4	4	1	4	6613.2864	0.0051
5	2	3	4	1	4	6613.2864	0.0051
4	3	1	3	2	1	7399.5088	0.0027
4	3	2	3	2	1	7399.5088	0.0027
4	3	1	3	2	2	7399.5088	0.0027
4	3	2	3	2	2	7399.5088	0.0027
6	2	5	5	1	4	7448.2442	-0.0043
6	2	4	5	1	4	7448.2442	-0.0043
6	2	5	5	1	5	7449.3096	-0.0032
6	2	4	5	1	5	7449.3096	-0.0032
11	2	10	11	1	10	2431.3105	0.0010
10	2	9	10	1	9	2431.6908	-0.0088
9	2	8	9	1	8	2432.0532	-0.0013
8	2	7	8	1	7	2432.3731	-0.0007
7	2	6	7	1	6	2432.6573	-0.0004
6	2	5	6	1	5	2432.9063	0.0002
5	2	4	5	1	4	2433.1213	0.0022
4	2	3	4	1	3	2433.3036	0.0071
3	2	2	3	1	2	2433.4616	0.0232
2	2	1	2	1	1	2433.5520	0.0071
2	2	0	2	1	2	2433.7566	-0.0011
3	2	1	3	1	3	2433.8571	-0.0071
4	2	2	4	1	4	2434.0081	0.0018
5	2	3	5	1	5	2434.1849	0.0010
6	2	4	6	1	6	2434.3973	0.0004
7	2	5	7	1	7	2434.6454	-0.0002
8	2	6	8	1	8	2434.9306	0.0006
9	2	7	9	1	9	2435.2487	-0.0012
10	2	8	10	1	10	2435.6051	-0.0005
11	2	9	11	1	11	2435.9943	-0.0028
12	2	10	12	1	12	2436.4243	-0.0001
13	2	11	13	1	13	2436.8883	0.0006
14	2	12	14	1	14	2437.3862	-0.0005
15	2	13	15	1	15	2437.9207	-0.0012
17	3	15	17	2	15	4056.0798	0.0071
16	3	14	16	2	14	4056.0798	0.0044
15	3	13	15	2	13	4056.0798	0.0021
14	3	12	14	2	12	4056.0798	0.0003
13	3	11	13	2	11	4056.0798	-0.0011
12	3	10	12	2	10	4056.0798	-0.0023
11	3	9	11	2	9	4056.0798	-0.0033
10	3	8	10	2	8	4056.0798	-0.0040
9	3	7	9	2	7	4056.0798	-0.0046
8	3	6	8	2	6	4056.0798	-0.0050
7	3	5	7	2	5	4056.0798	-0.0052
6	3	4	6	2	4	4056.0798	-0.0054
5	3	3	5	2	3	4056.0798	-0.0055
4	3	2	4	2	2	4056.0798	-0.0056
3	3	0	3	2	2	4056.0798	-0.0057
3	3	1	3	2	1	4056.0798	-0.0056
4	3	1	4	2	3	4056.0798	-0.0057
5	3	2	5	2	4	4056.0798	-0.0057
6	3	3	6	2	5	4056.0798	-0.0057
7	3	4	7	2	6	4056.0798	-0.0058
8	3	5	8	2	7	4056.0798	-0.0059
9	3	6	9	2	8	4056.0798	-0.0061
10	3	7	10	2	9	4056.0798	-0.0063
11	3	8	11	2	10	4056.0798	-0.0066
12	3	9	12	2	11	4056.0798	-0.0070
13	3	10	13	2	12	4056.0798	-0.0075
14	3	11	14	2	13	4056.0798	-0.0081

Table S12. Continued.

J'	K_a'	K_c'	J''	K_a''	K_c''	Obs	Res
15	3	12	15	2	14	4056.0798	-0.0089
16	3	13	16	2	15	4056.0798	-0.0098
17	3	14	17	2	16	4056.0798	-0.0109
18	4	15	18	3	15	5678.5193	0.0009
17	4	14	17	3	14	5678.5193	0.0006
16	4	13	16	3	13	5678.5193	0.0004
15	4	12	15	3	12	5678.5193	0.0002
14	4	11	14	3	11	5678.5193	0.0001
13	4	10	13	3	10	5678.5193	0.0000
12	4	9	12	3	9	5678.5193	-0.0001
11	4	8	11	3	8	5678.5193	-0.0002
10	4	7	10	3	7	5678.5193	-0.0002
9	4	6	9	3	6	5678.5193	-0.0003
8	4	5	8	3	5	5678.5193	-0.0003
7	4	4	7	3	4	5678.5193	-0.0003
6	4	3	6	3	3	5678.5193	-0.0003
5	4	2	5	3	2	5678.5193	-0.0003
4	4	1	4	3	1	5678.5193	-0.0003
4	4	0	4	3	2	5678.5193	-0.0003
5	4	1	5	3	3	5678.5193	-0.0003
6	4	2	6	3	4	5678.5193	-0.0003
7	4	3	7	3	5	5678.5193	-0.0003
8	4	4	8	3	6	5678.5193	-0.0003
9	4	5	9	3	7	5678.5193	-0.0003
10	4	6	10	3	8	5678.5193	-0.0002
11	4	7	11	3	9	5678.5193	-0.0002
12	4	8	12	3	10	5678.5193	-0.0001
13	4	9	13	3	11	5678.5193	0.0000
14	4	10	14	3	12	5678.5193	0.0001
15	4	11	15	3	13	5678.5193	0.0002
16	4	12	16	3	14	5678.5193	0.0004
17	4	13	17	3	15	5678.5193	0.0006
18	4	14	18	3	16	5678.5193	0.0009
18	5	14	18	4	14	7300.9444	-0.0089
17	5	13	17	4	13	7300.9444	-0.0090
15	5	11	15	4	11	7300.9444	-0.0092
16	5	12	16	4	12	7300.9444	-0.0091
14	5	10	14	4	10	7300.9444	-0.0093
13	5	9	13	4	9	7300.9444	-0.0093
12	5	8	12	4	8	7300.9444	-0.0094
11	5	7	11	4	7	7300.9444	-0.0094
10	5	6	10	4	6	7300.9444	-0.0094
9	5	5	9	4	5	7300.9444	-0.0094
8	5	4	8	4	4	7300.9444	-0.0094
7	5	3	7	4	3	7300.9444	-0.0095
6	5	2	6	4	2	7300.9444	-0.0095
5	5	1	5	4	1	7300.9444	-0.0095
5	5	0	5	4	2	7300.9444	-0.0095
6	5	1	6	4	3	7300.9444	-0.0095
7	5	2	7	4	4	7300.9444	-0.0095
8	5	3	8	4	5	7300.9444	-0.0094
9	5	4	9	4	6	7300.9444	-0.0094
10	5	5	10	4	7	7300.9444	-0.0094
11	5	6	11	4	8	7300.9444	-0.0094
12	5	7	12	4	9	7300.9444	-0.0094
13	5	8	13	4	10	7300.9444	-0.0093
14	5	9	14	4	11	7300.9444	-0.0093
15	5	10	15	4	12	7300.9444	-0.0092
16	5	11	16	4	13	7300.9444	-0.0091
17	5	12	17	4	14	7300.9444	-0.0090
18	5	13	18	4	15	7300.9444	-0.0089

Table S13. Observed frequencies and residuals (MHz) for the ^{13}C isotopologues of ATE for $J'K_a'K_c' \leftarrow J''K_a''K_c''$ transitions using the SPFIT program.

J'	K_a'	K_c'	J''	K_a''	K_c''	Obs	Res
						$^{13}C_2$	
4	1	3	3	0	3	4154.7051	0.0008
5	1	4	4	0	4	4990.7067	0.0000
4	2	2	3	1	2	5776.2053	-0.0016
4	2	3	3	1	3	5776.5587	-0.0031
3	3	0	2	2	0	6562.5054	0.0001
3	3	1	2	2	1	6562.5054	0.0001
4	3	2	3	2	2	7398.3640	0.0038
4	3	1	3	2	1	7398.3640	0.0038
12	4	9	12	3	9	5676.9146	-0.0008
11	4	8	11	3	8	5676.9146	-0.0008
10	4	7	10	3	7	5676.9146	-0.0008
9	4	6	9	3	6	5676.9146	-0.0008
8	4	5	8	3	5	5676.9146	-0.0008
7	4	4	7	3	4	5676.9146	-0.0008
6	4	3	6	3	3	5676.9146	-0.0008
5	4	2	5	3	2	5676.9146	-0.0008
4	4	1	4	3	1	5676.9146	-0.0008
4	4	0	4	3	2	5676.9146	-0.0008
5	4	1	5	3	3	5676.9146	-0.0008
6	4	2	6	3	4	5676.9146	-0.0008
7	4	3	7	3	5	5676.9146	-0.0008
8	4	4	8	3	6	5676.9146	-0.0008
9	4	5	9	3	7	5676.9146	-0.0008
10	4	6	10	3	8	5676.9146	-0.0008
11	4	7	11	3	9	5676.9146	-0.0008
12	4	8	12	3	10	5676.9146	-0.0008
						$^{13}C_3$	
2	2	1	1	1	1	4090.5768	0.0083
4	1	3	3	0	3	4150.8305	0.0026
5	1	4	4	0	4	4987.6838	-0.0023
3	3	1	2	2	1	6538.6569	-0.0046
3	3	0	2	2	0	6538.6569	-0.0046
4	3	2	3	2	2	7373.9329	-0.0004
4	3	1	3	2	1	7373.9329	-0.0004
12	4	9	12	3	9	5645.9777	0.0001
11	4	8	11	3	8	5645.9777	0.0001
10	4	7	10	3	7	5645.9777	0.0001
9	4	6	9	3	6	5645.9777	0.0001
8	4	5	8	3	5	5645.9777	0.0001
7	4	4	7	3	4	5645.9777	0.0001
6	4	3	6	3	3	5645.9777	0.0001
5	4	2	5	3	2	5645.9777	0.0001
4	4	1	4	3	1	5645.9777	0.0001
4	4	0	4	3	2	5645.9777	0.0001
5	4	1	5	3	3	5645.9777	0.0001
6	4	2	6	3	4	5645.9777	0.0001
7	4	3	7	3	5	5645.9777	0.0001
8	4	4	8	3	6	5645.9777	0.0001
9	4	5	9	3	7	5645.9777	0.0001
10	4	6	10	3	8	5645.9777	0.0001
11	4	7	11	3	9	5645.9777	0.0001
12	4	8	12	3	10	5645.9777	0.0001
						$^{13}C_4$	
5	1	4	4	0	4	4977.3927	0.0000
3	3	1	2	2	1	6528.7671	-0.0005
3	3	0	2	2	0	6528.7671	-0.0005
4	3	2	3	2	2	7361.8224	0.0004
4	3	1	3	2	1	7361.8224	0.0004
12	4	9	12	3	9	5641.4348	0.0001
11	4	8	11	3	8	5641.4348	0.0001
10	4	7	10	3	7	5641.4348	0.0001
9	4	6	9	3	6	5641.4348	0.0001
8	4	5	8	3	5	5641.4348	0.0001
7	4	4	7	3	4	5641.4348	0.0001
6	4	3	6	3	3	5641.4348	0.0001
5	4	2	5	3	2	5641.4348	0.0001
4	4	1	4	3	1	5641.4348	0.0001
4	4	0	4	3	2	5641.4348	0.0001
5	4	1	5	3	3	5641.4348	0.0001
6	4	2	6	3	4	5641.4348	0.0001
7	4	3	7	3	5	5641.4348	0.0001
8	4	4	8	3	6	5641.4348	0.0001
9	4	5	9	3	7	5641.4348	0.0001
10	4	6	10	3	8	5641.4348	0.0001
11	4	7	11	3	9	5641.4348	0.0001
12	4	8	12	3	10	5641.4348	0.0001
						$^{13}C_5$	
4	1	3	3	0	3	4134.9112	-0.0041
3	2	1	2	1	1	4914.7755	0.0234
5	1	4	4	0	4	4967.6297	0.0062
4	2	3	3	1	3	5748.8969	-0.0119
3	3	0	2	2	0	6531.2897	0.0015
3	3	1	2	2	1	6531.2897	0.0015
5	2	3	4	1	3	6573.9920	-0.0078
5	2	4	4	1	4	6581.2518	0.0011
4	3	1	3	2	1	7362.1748	-0.0022
4	3	2	3	2	2	7362.1748	-0.0022
12	4	9	12	3	9	5654.0639	0.0026
11	4	8	11	3	8	5654.0639	0.0026

Table S13. Continued.

J'	K_a'	K_c'	J''	K_a''	K_c''	Obs	Res
10	4	7	10	3	7	5654.0639	0.0026
9	4	6	9	3	6	5654.0639	0.0026
8	4	5	8	3	5	5654.0639	0.0026
7	4	4	7	3	4	5654.0639	0.0026
6	4	3	6	3	3	5654.0639	0.0026
5	4	2	5	3	2	5654.0639	0.0026
4	4	1	4	3	1	5654.0638	-0.0045
4	4	0	4	3	2	5654.0638	-0.0045
5	4	1	5	3	3	5654.0639	0.0026
6	4	2	6	3	4	5654.0639	0.0026
7	4	3	7	3	5	5654.0639	0.0026
8	4	4	8	3	6	5654.0639	0.0026
9	4	5	9	3	7	5654.0639	0.0026
10	4	6	10	3	8	5654.0639	0.0026
11	4	7	11	3	9	5654.0639	0.0026
12	4	8	12	3	10	5654.0639	0.0026
						$^{13}C_6$	
3	2	2	2	1	2	4920.4626	0.0025
4	2	2	3	1	2	5749.9999	0.0012
3	3	0	2	2	0	6535.6731	-0.0022
3	3	1	2	2	1	6535.6731	-0.0022
4	3	2	3	2	2	7367.5647	-0.0015
4	3	1	3	2	1	7367.5647	-0.0015
12	4	9	12	3	9	5655.9994	0.0010
11	4	8	11	3	8	5655.9994	0.0010
10	4	7	10	3	7	5655.9994	0.0010
9	4	6	9	3	6	5655.9994	0.0010
8	4	5	8	3	5	5655.9994	0.0010
7	4	4	7	3	4	5655.9994	0.0010
6	4	3	6	3	3	5655.9994	0.0010
5	4	2	5	3	2	5655.9994	0.0010
4	4	1	4	3	1	5655.9994	0.0010
4	4	0	4	3	2	5655.9994	0.0010
5	4	1	5	3	3	5655.9994	0.0010
6	4	2	6	3	4	5655.9994	0.0010
7	4	3	7	3	5	5655.9994	0.0010
8	4	4	8	3	6	5655.9994	0.0010
9	4	5	9	3	7	5655.9994	0.0010
10	4	6	10	3	8	5655.9994	0.0010
11	4	7	11	3	9	5655.9994	0.0010
12	4	8	12	3	10	5655.9994	0.0010
						$^{13}C_7$	
4	1	3	3	0	3	4133.8782	-0.0019
5	1	4	4	0	4	4965.8647	0.0012
3	3	0	2	2	0	6536.5903	-0.0026
3	3	1	2	2	1	6536.5903	-0.0026
4	3	1	3	2	1	7367.0212	0.0022
4	3	2	3	2	2	7367.0212	0.0022
12	4	9	12	3	9	5663.4336	0.0003
11	4	8	11	3	8	5663.4336	0.0003
10	4	7	10	3	7	5663.4336	0.0003
9	4	6	9	3	6	5663.4336	0.0003
8	4	5	8	3	5	5663.4336	0.0003
7	4	4	7	3	4	5663.4336	0.0003
6	4	3	6	3	3	5663.4336	0.0003
5	4	2	5	3	2	5663.4336	0.0003
4	4	1	4	3	1	5663.4336	0.0003
4	4	0	4	3	2	5663.4336	0.0003
5	4	1	5	3	3	5663.4336	0.0003
6	4	2	6	3	4	5663.4336	0.0003
7	4	3	7	3	5	5663.4336	0.0003
8	4	4	8	3	6	5663.4336	0.0003
9	4	5	9	3	7	5663.4336	0.0003
10	4	6	10	3	8	5663.4336	0.0003
11	4	7	11	3	9	5663.4336	0.0003
12	4	8	12	3	10	5663.4336	0.0003
						$^{13}C_8$	
4	1	3	3	0	3	4140.8907	0.0011
3	2	1	2	1	1	4916.2459	-0.0146
3	2	2	2	1	2	4918.5023	-0.0083
5	1	4	4	0	4	4975.4357	-0.0020
3	3	1	2	2	1	6530.3168	0.0135
3	3	0	2	2	0	6530.3168	0.0135
4	3	1	3	2	1	7362.9800	0.0085
4	3	2	3	2	2	7362.9800	0.0085
12	4	9	12	3	9	5645.2019	-0.0057
11	4	8	11	3	8	5645.2019	-0.0057
10	4	7	10	3	7	5645.2019	-0.0057
9	4	6	9	3	6	5645.2019	-0.0057
8	4	5	8	3	5	5645.2019	-0.0057
7	4	4	7	3	4	5645.2019	-0.0057
6	4	3	6	3	3	5645.2019	-0.0057
5	4	2	5	3	2	5645.2019	-0.0057
4	4	1	4	3	1	5645.2019	-0.0057
4	4	0	4	3	2	5645.2019	-0.0057
5	4	1	5	3	3	5645.2019	-0.0057
6	4	2	6	3	4	5645.2019	-0.0057
7	4	3	7	3	5	5645.2019	-0.0057
8	4	4	8	3	6	5645.2019	-0.0057
9	4	5	9	3	7	5645.2019	-0.0057

Table S13. Continued.

J'	K_a'	K_c'	J''	K_a''	K_c''	Obs	Res
10	4	6	10	3	8	5645.2019	-0.0057
11	4	7	11	3	9	5645.2019	-0.0057
12	4	8	12	3	10	5645.2019	-0.0057
						$^{13}C_9$	
3	1	2	2	0	2	3313.0237	-0.0016
2	2	0	1	1	0	4086.5522	-0.0084
2	2	1	1	1	1	4087.3045	0.0077
4	1	3	3	0	3	4149.5683	-0.0049
5	1	4	4	0	4	4986.4943	0.0034
4	2	3	3	1	3	5759.2888	0.0032
6	1	5	5	0	5	5823.7768	-0.0028
3	3	1	2	2	1	6533.1946	0.0053
3	3	0	2	2	0	6533.1946	0.0053
7	1	6	6	0	6	6661.4424	0.0011
4	3	1	3	2	1	7368.2602	-0.0027
4	3	2	3	2	2	7368.2602	-0.0027
13	4	10	13	3	10	5639.1443	-0.0022
12	4	9	12	3	9	5639.1443	-0.0022
11	4	8	11	3	8	5639.1443	-0.0022
10	4	7	10	3	7	5639.1443	-0.0022
9	4	6	9	3	6	5639.1443	-0.0022
8	4	5	8	3	5	5639.1443	-0.0022
7	4	4	7	3	4	5639.1443	-0.0022
6	4	3	6	3	3	5639.1443	-0.0022
5	4	2	5	3	2	5639.1443	-0.0022
4	4	1	4	3	1	5639.1443	-0.0022
4	4	0	4	3	2	5639.1443	-0.0022
5	4	1	5	3	3	5639.1443	-0.0022
6	4	2	6	3	4	5639.1443	-0.0022
7	4	3	7	3	5	5639.1443	-0.0022
8	4	4	8	3	6	5639.1443	-0.0022
9	4	5	9	3	7	5639.1443	-0.0022
10	4	6	10	3	8	5639.1443	-0.0022
11	4	7	11	3	9	5639.1443	-0.0022
12	4	8	12	3	10	5639.1443	-0.0022
13	4	10	13	3	10	5639.1443	-0.0022
						$^{13}C_{10}$	
3	2	1	2	1	1	4910.9565	0.0153
3	2	2	2	1	2	4913.4029	0.0153
3	3	0	2	2	0	6523.4461	-0.0120
3	3	1	2	2	1	6523.4461	-0.0120
4	3	1	3	2	1	7355.1837	-0.0140
4	3	2	3	2	2	7355.1837	-0.0140
12	4	9	12	3	9	5639.5295	0.0054
11	4	8	11	3	8	5639.5295	0.0054
10	4	7	10	3	7	5639.5295	0.0054
9	4	6	9	3	6	5639.5295	0.0054
8	4	5	8	3	5	5639.5295	0.0054
7	4	4	7	3	4	5639.5295	0.0054
6	4	3	6	3	3	5639.5295	0.0054
5	4	2	5	3	2	5639.5295	0.0054
4	4	1	4	3	1	5639.5295	0.0054
4	4	0	4	3	2	5639.5295	0.0054
5	4	1	5	3	3	5639.5295	0.0054
6	4	2	6	3	4	5639.5295	0.0054
7	4	3	7	3	5	5639.5295	0.0054
8	4	4	8	3	6	5639.5295	0.0054
9	4	5	9	3	7	5639.5295	0.0054
10	4	6	10	3	8	5639.5295	0.0054
11	4	7	11	3	9	5639.5295	0.0054
12	4	8	12	3	10	5639.5295	0.0054
						$^{13}C_{11}$	
3	2	1	2	1	1	4915.5057	-0.0015
4	2	2	3	1	2	5748.8974	0.0007
3	3	1	2	2	1	6525.3193	0.0037
3	3	0	2	2	0	6525.3193	0.0037
4	3	2	3	2	2	7359.9688	-0.0024
4	3	1	3	2	1	7359.9688	-0.0024
12	4	9	12	3	9	5629.8763	-0.0006
11	4	8	11	3	8	5629.8763	-0.0006
10	4	7	10	3	7	5629.8763	-0.0006
9	4	6	9	3	6	5629.8763	-0.0006
8	4	5	8	3	5	5629.8763	-0.0006
7	4	4	7	3	4	5629.8763	-0.0006
6	4	3	6	3	3	5629.8763	-0.0006
5	4	2	5	3	2	5629.8763	-0.0006
4	4	1	4	3	1	5629.8763	-0.0006
4	4	0	4	3	2	5629.8763	-0.0006
5	4	1	5	3	3	5629.8763	-0.0006
6	4	2	6	3	4	5629.8763	-0.0006
7	4	3	7	3	5	5629.8763	-0.0006
8	4	4	8	3	6	5629.8763	-0.0006
9	4	5	9	3	7	5629.8763	-0.0006
10	4	6	10	3	8	5629.8763	-0.0006
11	4	7	11	3	9	5629.8763	-0.0006
12	4	8	12	3	10	5629.8763	-0.0006
						$^{13}C_{12}$	
4	1	3	3	0	3	4138.2761	-0.0032
5	1	4	4	0	4	4969.5249	0.0000
4	2	3	3	1	3	5765.4372	0.0053

Table S13. Continued.

J'	K_a'	K_c'	J''	K_a''	K_c''	Obs	Res
3	3	1	2	2	1	6561.4437	-0.0005
3	3	0	2	2	0	6561.4444	0.0001
4	3	2	3	2	2	7392.5124	-0.0018
4	3	1	3	2	1	7392.5124	-0.0018
12	4	9	12	3	9	5695.5267	-0.0002
11	4	8	11	3	8	5695.5267	-0.0002
10	4	7	10	3	7	5695.5267	-0.0002
9	4	6	9	3	6	5695.5267	-0.0002
8	4	5	8	3	5	5695.5267	-0.0002
7	4	4	7	3	4	5695.5267	-0.0002
6	4	3	6	3	3	5695.5267	-0.0002
5	4	2	5	3	2	5695.5267	-0.0002
4	4	1	4	3	1	5695.5267	-0.0002
4	4	0	4	3	2	5695.5267	-0.0002
5	4	1	5	3	3	5695.5267	-0.0002
6	4	2	6	3	4	5695.5267	-0.0002
7	4	3	7	3	5	5695.5267	-0.0002
8	4	4	8	3	6	5695.5267	-0.0002
9	4	5	9	3	7	5695.5267	-0.0002
10	4	6	10	3	8	5695.5267	-0.0002
11	4	7	11	3	9	5695.5267	-0.0002
12	4	8	12	3	10	5695.5267	-0.0002
						$^{13}C_{13}$	
5	1	4	4	0	4	4963.5091	0.0000
3	3	0	2	2	0	6525.5173	-0.0127
3	3	1	2	2	1	6525.5173	-0.0127
4	3	1	3	2	1	7355.7397	0.0095
4	3	2	3	2	2	7355.7397	0.0095
12	4	9	12	3	9	5648.8955	0.0044
11	4	8	11	3	8	5648.8955	0.0044
10	4	7	10	3	7	5648.8955	0.0044
9	4	6	9	3	6	5648.8955	0.0044
8	4	5	8	3	5	5648.8955	0.0044
7	4	4	7	3	4	5648.8955	0.0044
6	4	3	6	3	3	5648.8955	0.0044
5	4	2	5	3	2	5648.8955	0.0044
4	4	1	4	3	1	5648.8961	-0.0021
4	4	0	4	3	2	5648.8961	-0.0021
5	4	1	5	3	3	5648.8955	0.0044
6	4	2	6	3	4	5648.8955	0.0044
7	4	3	7	3	5	5648.8955	0.0044
8	4	4	8	3	6	5648.8955	0.0044
9	4	5	9	3	7	5648.8955	0.0044
10	4	6	10	3	8	5648.8955	0.0044
11	4	7	11	3	9	5648.8955	0.0044
12	4	8	12	3	10	5648.8955	0.0044
						$^{13}C_{14}$	
3	3	0	2	2	0	6542.1056	0.0000
3	3	1	2	2	1	6542.1064	0.0006
5	2	4	4	1	4	6570.4655	0.0000
4	3	2	3	2	2	7368.1275	-0.0004
4	3	1	3	2	1	7368.1275	-0.0004
12	4	9	12	3	9	5689.6506	-0.0001
11	4	8	11	3	8	5689.6506	-0.0001
10	4	7	10	3	7	5689.6506	-0.0001
9	4	6	9	3	6	5689.6506	-0.0001
8	4	5	8	3	5	5689.6506	-0.0001
7	4	4	7	3	4	5689.6506	-0.0001
6	4	3	6	3	3	5689.6506	-0.0001
5	4	2	5	3	2	5689.6506	-0.0001
4	4	1	4	3	1	5689.6506	-0.0001
4	4	0	4	3	2	5689.6506	-0.0001
5	4	1	5	3	3	5689.6506	-0.0001
6	4	2	6	3	4	5689.6506	-0.0001
7	4	3	7	3	5	5689.6506	-0.0001
8	4	4	8	3	6	5689.6506	-0.0001
9	4	5	9	3	7	5689.6506	-0.0001
10	4	6	10	3	8	5689.6506	-0.0001
11	4	7	11	3	9	5689.6506	-0.0001
12	4	8	12	3	10	5689.6506	-0.0001
						$^{13}C_{15}$	
4	1	3	3	0	3	4128.5071	-0.0157
5	1	4	4	0	4	4959.7535	0.0104
3	3	1	2	2	1	6526.6458	0.0133
3	3	0	2	2	0	6526.6458	0.0133
4	3	2	3	2	2	7355.7395	-0.0074
4	3	1	3	2	1	7355.7395	-0.0074
12	4	9	12	3	9	5654.9959	0.0031
11	4	8	11	3	8	5654.9959	0.0031
10	4	7	10	3	7	5654.9959	0.0031
9	4	6	9	3	6	5654.9959	0.0031
8	4	5	8	3	5	5654.9959	0.0031
7	4	4	7	3	4	5654.9959	0.0031
6	4	3	6	3	3	5654.9959	0.0031
5	4	2	5	3	2	5654.9959	0.0031
4	4	1	4	3	1	5654.9955	-0.0066
4	4	0	4	3	2	5654.9955	-0.0066
5	4	1	5	3	3	5654.9959	0.0031
6	4	2	6	3	4	5654.9959	0.0031
7	4	3	7	3	5	5654.9959	0.0031

Table S13. Continued.

J'	K_a'	K_c'	J''	K_a''	K_c''	Obs	Res
8	4	4	8	3	6	5654.9959	0.0031
9	4	5	9	3	7	5654.9959	0.0031
10	4	6	10	3	8	5654.9959	0.0031
11	4	7	11	3	9	5654.9959	0.0031
12	4	8	12	3	10	5654.9959	0.0031

Table S14. Observed frequencies and residuals (MHz) for the parent species of AMES for $J'K'_aK'_c \leftarrow J''K''_aK''_c$ transitions using the XIAM program.

J'	K'_a	K'_c	J''	K''_a	K''_c	S	Obs	Res
2	0	2	1	0	1	A	2030.2516	-0.0194
2	0	2	1	0	1	E	2030.2516	0.0058
2	1	1	1	1	0	A	2057.1759	-0.0007
2	1	1	1	1	0	E	2057.0166	-0.0075
3	1	3	2	1	2	A	3006.4284	0.0239
3	1	3	2	1	2	E	3006.4284	-0.0171
3	0	3	2	0	2	A	3043.9825	-0.0004
3	0	3	2	0	2	E	3043.9825	0.0354
3	2	2	2	2	1	A	3046.2389	-0.0214
3	2	2	2	2	1	E	3047.2106	0.0005
3	1	2	2	1	1	A	3085.3804	-0.0237
3	1	2	2	1	1	E	3085.3804	0.0077
4	1	4	3	1	3	A	4007.9051	0.0142
4	1	4	3	1	3	E	4007.9051	-0.0047
4	0	4	3	0	3	A	4056.0010	0.0042
4	2	3	3	2	2	A	4061.2284	-0.0072
4	2	3	3	2	2	E	4062.7161	-0.0008
4	3	1	3	3	0	A	4062.8128	-0.0291
4	3	1	3	3	0	E	4062.8128	0.0001
4	3	2	3	3	1	A	4062.8128	0.0172
4	3	2	3	3	1	E	4062.8128	-0.0016
4	2	2	3	2	1	A	4066.9186	0.0001
4	2	2	3	2	1	E	4065.4321	0.0015
4	1	3	3	1	2	A	4113.1987	0.0111
4	1	3	3	1	2	E	4113.1588	-0.0197
5	1	5	4	1	4	A	5008.8501	0.0059
5	1	5	4	1	4	E	5008.8501	-0.0095
5	0	5	4	0	4	A	5065.7861	0.0064
5	2	4	4	2	3	A	5075.8276	-0.0023
5	2	4	4	2	3	E	5076.9711	0.0039
5	4	2	4	4	1	A	5078.3663	0.0046
5	4	2	4	4	1	E	5078.3663	0.0108
5	4	1	4	4	0	A	5078.3663	0.0039
5	4	1	4	4	0	E	5078.3663	0.0121
5	3	3	4	3	2	A	5078.9739	-0.0012
5	3	3	4	3	2	E	5079.0570	0.0065
5	3	2	4	3	1	A	5079.1370	0.0001
5	3	2	4	3	1	E	5079.0570	0.0084
5	2	3	4	2	2	A	5087.1448	-0.0024
5	2	3	4	2	2	E	5086.0009	-0.0012
5	1	4	4	1	3	A	5140.3604	-0.0006
5	1	4	4	1	3	E	5140.3604	0.0061
6	1	6	5	1	5	A	6009.1648	0.0060
6	1	6	5	1	5	E	6009.1648	-0.0137
6	0	6	5	0	5	A	6072.8539	-0.0026
6	2	5	5	2	4	A	6089.9437	-0.0048
6	2	5	5	2	4	E	6090.5263	0.0038
6	5	2	5	5	1	A	6093.9370	0.0020
6	5	2	5	5	1	E	6093.9370	0.0103
6	5	1	5	5	0	A	6093.9370	0.0019
6	5	1	5	5	0	E	6093.9370	0.0116
6	4	3	5	4	2	A	6094.4503	0.0026
6	4	3	5	4	2	E	6094.4503	0.0086
6	4	2	5	4	1	A	6094.4503	-0.0005
6	4	2	5	4	1	E	6094.4503	0.0109
6	3	4	5	3	3	A	6095.4363	0.0022
6	3	4	5	3	3	E	6095.6513	0.0130
6	3	3	5	3	2	A	6095.8693	0.0044
6	3	3	5	3	2	E	6095.6513	0.0064
6	2	4	5	2	3	A	6109.5940	-0.0090
6	2	4	5	2	3	E	6109.0132	-0.0067
6	1	5	5	1	4	A	6166.7392	-0.0030
6	1	5	5	1	4	E	6166.7392	0.0094
7	1	7	6	1	6	A	7008.7386	-0.0082
7	1	7	6	1	6	E	7008.7386	-0.0387
7	0	7	6	0	6	A	7076.8513	-0.0044
7	2	6	6	2	5	A	7103.4981	0.0009
7	2	6	6	2	5	E	7103.7757	0.0083
7	5	3	6	5	2	A	7109.9507	0.0042
7	5	3	6	5	2	E	7109.9507	0.0138
7	5	2	6	5	1	A	7109.9507	0.0042
7	5	2	6	5	1	E	7109.9507	0.0157
7	4	4	6	4	3	A	7110.7612	0.0034
7	4	4	6	4	3	E	7110.7612	0.0066
7	4	3	6	4	2	A	7110.7612	-0.0070
7	4	3	6	4	2	E	7110.7612	0.0101
7	3	5	6	3	4	A	7112.1920	0.0037
7	3	5	6	3	4	E	7112.6439	0.0124
7	3	4	6	3	3	A	7113.1597	0.0036
7	3	4	6	3	3	E	7112.7013	0.0068
7	2	5	6	2	4	A	7134.5664	0.0062
7	2	5	6	2	4	E	7134.2870	0.0068
7	1	6	6	1	5	A	7192.1301	0.0010
7	1	6	6	1	5	E	7192.1301	0.0238
5	1	5	4	2	2	A	2129.6050	-0.0129
5	1	5	4	2	2	E	2127.3544	-0.0163
3	0	3	2	1	2	A	2170.2491	-0.0037
7	2	6	6	3	3	A	2528.9829	-0.0058
5	1	4	4	2	3	A	2532.9663	-0.0024
5	1	4	4	2	3	E	2535.4606	-0.0326

Table S14. Continued.

J'	K_a'	K_c'	J''	K_a''	K_c''	S	Obs	Res
7	2	5	6	3	4	A	2600.2018	0.0012
9	3	7	8	4	4	A	2763.8216	-0.0012
9	3	6	8	4	5	A	2770.9358	0.0034
2	1	2	1	0	1	A	2904.0026	0.0014
2	1	2	1	0	1	E	2903.8797	-0.0174
6	1	6	5	2	3	A	3051.6202	-0.0093
6	1	6	5	2	3	E	3050.5133	-0.0339
4	0	4	3	1	3	A	3219.8460	0.0010
8	2	7	7	3	4	A	3532.2099	-0.0062
6	1	5	5	2	4	A	3623.8821	0.0011
6	1	5	5	2	4	E	3625.2270	-0.0288
8	2	6	7	3	5	A	3650.1469	-0.0012
8	2	6	7	3	5	E	3657.4042	0.0038
3	1	3	2	0	2	A	3880.1385	0.0038
3	1	3	2	0	2	E	3880.0602	-0.0365
7	1	7	6	2	4	A	3950.7670	-0.0064
7	1	7	6	2	4	E	3950.2765	-0.0281
5	0	5	4	1	4	A	4277.7331	-0.0006
9	2	7	8	3	6	A	4713.1711	0.0155
7	1	6	6	2	5	A	4726.0620	0.0003
7	1	6	6	2	5	E	4726.8241	-0.0156
2	2	1	1	1	0	A	4755.6669	0.0024
2	2	1	1	1	0	E	4750.5405	0.0312
2	2	0	1	1	1	A	4782.5713	0.0011
2	2	0	1	1	1	E	4787.4683	-0.0272
8	1	8	7	2	5	A	4823.7559	0.0028
4	1	4	3	0	3	A	4844.0417	-0.0011
4	1	4	3	0	3	E	4844.0417	-0.0178
6	0	6	5	1	5	A	5341.7536	0.0074
10	2	9	9	3	6	A	5519.2983	-0.0081
9	1	9	8	2	6	A	5667.1104	0.0013
3	2	2	2	1	1	A	5744.7514	0.0031
3	2	2	2	1	1	E	5740.6960	0.0007
10	2	8	9	3	7	A	5791.1846	0.0114
10	2	8	9	3	7	E	5796.2905	-0.0312
5	1	5	4	0	4	A	5796.8882	-0.0019
3	2	1	2	1	2	A	5826.6163	0.0131
3	2	1	2	1	2	E	5830.4094	-0.0042
8	1	7	7	2	6	A	5838.8638	0.0034
8	1	7	7	2	6	E	5839.3487	0.0163
7	0	7	6	1	6	A	6409.4419	-0.0013
10	1	10	9	2	7	A	6477.4573	0.0133
11	2	10	10	3	7	A	6499.3889	-0.0102
4	2	3	3	1	2	A	6720.5847	0.0048
4	2	3	3	1	2	E	6718.0718	0.0322
6	1	6	5	0	5	A	6740.2372	-0.0320
4	2	2	3	1	3	A	6887.1210	0.0039
4	2	2	3	1	3	E	6889.3977	-0.0009
9	1	8	8	2	7	A	6961.4740	0.0071
8	0	8	7	1	7	A	7478.2676	0.0101
3	3	1	2	2	0	A	7609.3594	-0.0023
3	3	0	2	2	1	A	7609.9233	-0.0159
7	1	7	6	0	6	A	7676.1389	-0.0205
5	2	4	4	1	3	A	7683.2287	0.0064
5	2	4	4	1	3	E	7681.8612	0.0329
5	2	3	4	1	4	A	7966.3826	0.0091
5	2	3	4	1	4	E	7967.4993	0.0084
12	2	10	12	1	11	A	2289.8103	-0.0316
11	2	9	11	1	10	A	2291.4816	-0.0221
13	2	11	13	1	12	A	2309.7428	-0.0303
10	2	8	10	1	9	A	2311.7039	-0.0236
9	2	7	9	1	8	A	2347.0938	0.0012
9	2	7	9	1	8	E	2347.1343	-0.0428
8	2	6	8	1	7	A	2393.8495	0.0089
8	2	6	8	1	7	E	2393.9425	-0.0108
7	2	5	7	1	6	A	2448.0059	0.0051
7	2	5	7	1	6	E	2448.2361	0.0156
6	2	4	6	1	5	A	2505.5738	0.0040
6	2	4	6	1	5	E	2506.0792	0.0325
5	2	3	5	1	4	A	2562.7142	0.0052
5	2	3	5	1	4	E	2563.7950	0.0384
4	2	2	4	1	3	A	2615.9276	0.0047
4	2	2	4	1	3	E	2618.1403	0.0314
3	2	1	3	1	2	A	2662.2051	0.0132
3	2	1	3	1	2	E	2665.8695	0.0127
2	2	0	2	1	1	A	2699.0621	0.0043
2	2	0	2	1	1	E	2703.6428	-0.0034
2	2	1	2	1	2	A	2777.5019	0.0068
2	2	1	2	1	2	E	2772.6529	-0.0164
3	2	2	3	1	3	A	2817.3734	0.0225
3	2	2	3	1	3	E	2813.4403	0.0065
4	2	3	4	1	4	A	2870.7034	0.0078
4	2	3	4	1	4	E	2868.2442	0.0033
5	2	4	5	1	5	A	2937.6918	0.0104
5	2	4	5	1	5	E	2936.3657	0.0172
6	2	5	6	1	6	A	3018.4717	0.0005
6	2	5	6	1	6	E	3017.7262	0.0337
7	2	6	7	1	7	A	3113.2254	0.0038
8	2	7	8	1	8	A	3222.0624	-0.0025
9	2	8	9	1	9	A	3345.0794	-0.0101

Table S14. Continued.

J'	K_a'	K_c'	J''	K_a''	K_c''	S	Obs	Res
10	2	9	10	1	10	A	3482.3040	-0.0165
11	2	10	11	1	11	A	3633.6746	-0.0288
17	3	14	17	2	15	A	3748.2577	0.0088
14	3	11	14	2	12	A	4052.1082	-0.0084
13	3	10	13	2	11	A	4149.8311	-0.0102
12	3	9	12	2	10	A	4239.5849	-0.0086
12	3	9	12	2	10	E	4240.7776	0.0350
11	3	8	11	2	9	A	4318.8152	-0.0142
11	3	8	11	2	9	E	4320.8301	0.0332
10	3	7	10	2	8	A	4385.9758	0.0002
10	3	7	10	2	8	E	4389.1416	0.0296
9	3	6	9	2	7	A	4440.4669	0.0086
9	3	6	9	2	7	E	4444.9384	0.0106
8	3	5	8	2	6	A	4482.6303	-0.0000
8	3	5	8	2	6	E	4488.2320	0.0070
7	3	4	7	2	5	A	4513.6007	-0.0013
7	3	4	7	2	5	E	4519.8763	0.0021
6	3	3	6	2	4	A	4534.9955	-0.0107
6	3	3	6	2	4	E	4541.4508	-0.0091
5	3	2	5	2	3	A	4548.7490	0.0047
5	3	2	5	2	3	E	4554.8340	-0.0008
4	3	1	4	2	2	A	4556.7572	0.0026
4	3	1	4	2	2	E	4561.7771	-0.0112
3	3	0	3	2	1	A	4560.8194	-0.0118
3	3	0	3	2	1	E	4564.3893	-0.0168
3	3	1	3	2	2	A	4563.6482	-0.0230
3	3	1	3	2	2	E	4559.7126	-0.0121
4	3	2	4	2	3	A	4565.2338	0.0027
4	3	2	4	2	3	E	4559.8077	-0.0145
5	3	3	5	2	4	A	4568.3801	0.0038
5	3	3	5	2	4	E	4561.8943	-0.0111
6	3	4	6	2	5	A	4573.8676	0.0058
6	3	4	6	2	5	E	4567.0118	-0.0094
7	3	5	7	2	6	A	4582.5608	0.0078
7	3	5	7	2	6	E	4575.8761	-0.0092
8	3	6	8	2	7	A	4595.4150	0.0111
8	3	6	8	2	7	E	4589.3935	-0.0099
9	3	7	9	2	8	A	4613.4428	0.0080
9	3	7	9	2	8	E	4608.5553	0.0085
10	3	8	10	2	9	A	4637.7125	0.0062
10	3	8	10	2	9	E	4634.1524	0.0156
11	3	9	11	2	10	A	4669.3034	0.0090
11	3	9	11	2	10	E	4666.8853	0.0068
12	3	10	12	2	11	A	4709.2780	0.0133
12	3	10	12	2	11	E	4707.6702	0.0182
13	3	11	13	2	12	A	4758.6666	0.0185
13	3	11	13	2	12	E	4757.5718	0.0384
14	3	12	14	2	13	A	4818.3903	-0.0271
14	4	10	14	3	11	A	6281.6148	-0.0141
13	4	9	13	3	10	A	6312.3625	-0.0230
12	4	8	12	3	9	A	6335.5404	0.0048
11	4	7	11	3	8	A	6352.5640	-0.0058
11	4	7	11	3	8	E	6359.8501	-0.0189
10	4	6	10	3	7	A	6364.8042	-0.0021
10	4	6	10	3	7	E	6371.0541	-0.0196
14	4	11	14	3	12	A	6368.9447	0.0036
14	4	11	14	3	12	E	6360.8744	-0.0144
15	4	12	15	3	13	A	6369.7617	0.0121
13	4	10	13	3	11	A	6369.9408	0.0065
12	4	9	12	3	10	A	6372.0546	-0.0022
9	4	5	9	3	6	A	6373.3631	-0.0044
9	4	5	9	3	6	E	6378.3213	-0.0274
11	4	8	11	3	9	A	6374.7477	-0.0115
11	4	8	11	3	9	E	6366.9084	-0.0182
10	4	7	10	3	8	A	6377.6224	0.0057
10	4	7	10	3	8	E	6370.8001	-0.0199
8	4	4	8	3	5	A	6379.1904	0.0102
8	4	4	8	3	5	E	6383.0025	-0.0064
9	4	6	9	3	7	A	6380.3378	0.0146
9	4	6	9	3	7	E	6374.8022	-0.0139
8	4	5	8	3	6	A	6382.7012	0.0197
8	4	5	8	3	6	E	6378.3213	-0.0085
7	4	3	7	3	4	A	6383.0035	0.0147
7	4	3	7	3	4	E	6386.0510	0.0257
7	4	4	7	3	5	A	6384.5644	-0.0244
7	4	4	7	3	5	E	6381.0258	-0.0057
6	4	2	6	3	3	A	6385.3972	0.0206
6	4	2	6	3	3	E	6387.9722	0.0033
6	4	3	6	3	4	A	6385.9749	-0.0444
6	4	3	6	3	4	E	6382.9027	-0.0058
5	4	1	5	3	2	A	6386.8135	0.0228
5	4	1	5	3	2	E	6389.1450	-0.0294
5	4	2	5	3	3	A	6387.0341	0.0284
5	4	2	5	3	3	E	6384.0890	-0.0161
4	4	0	4	3	1	A	6387.5563	-0.0089
4	4	0	4	3	1	E	6389.8379	-0.0310
4	4	1	4	3	2	A	6387.6259	0.0068
4	4	1	4	3	2	E	6384.7810	-0.0190

Table S15. Observed frequencies and residuals (MHz) for the ^{13}C isotopologues of AMES for $J'K_a'K_c' \leftarrow J''K_a''K_c''$ transitions using the SPFIT program.

J'	K_a'	K_c'	J''	K_a''	K_c''	Obs	Res
						$^{13}C_2$	
2	1	2	1	0	1	2898.2868	0.0020
4	0	4	3	1	3	3204.2818	-0.0013
3	1	3	2	0	2	3871.0497	0.0041
4	0	4	3	0	3	4041.9248	0.0035
5	0	5	4	1	4	4258.5210	0.0006
2	2	1	1	1	0	4752.2233	0.0094
2	2	0	1	1	1	4778.9963	-0.0101
4	1	4	3	0	3	4831.6349	0.0090
5	1	5	4	1	4	4991.4851	0.0102
5	0	5	4	0	4	5048.2261	0.0012
6	0	6	5	1	5	5318.8946	0.0022
3	2	2	2	1	1	5737.8785	0.0057
5	1	5	4	0	4	5781.1789	-0.0005
3	2	1	2	1	2	5819.3864	0.0086
6	0	6	5	0	5	6051.8438	-0.0030
4	2	3	3	1	2	6710.3410	0.0067
6	1	6	5	0	5	6721.2803	-0.0036
4	2	2	3	1	3	6876.1279	-0.0173
3	3	1	2	2	0	7604.6823	-0.0172
3	3	0	2	2	1	7605.2889	0.0171
7	1	7	6	0	6	7653.8895	-0.0114
5	2	3	4	1	4	7951.5471	-0.0024
						$^{13}C_4$	
4	0	4	3	0	3	4055.6293	-0.0115
2	2	1	1	1	0	4755.3842	-0.0013
2	2	0	1	1	1	4782.3222	0.0143
4	1	4	3	0	3	4843.5857	0.0021
5	1	5	4	1	4	5008.3801	0.0145
6	0	6	5	1	5	5341.3294	-0.0026
5	1	5	4	0	4	5796.2905	-0.0177
3	2	1	2	1	2	5826.2823	0.0098
6	1	6	5	0	5	6739.5541	-0.0074
3	3	1	2	2	0	7608.9377	-0.0005
3	3	0	2	2	1	7609.5049	-0.0117
7	1	7	6	0	6	7675.3390	0.0136
						$^{13}C_5$=$^{13}C_{11}$	
2	1	2	1	0	1	2895.6710	0.0086
3	1	3	2	1	2	3004.3363	-0.0093
3	0	3	2	0	2	3041.0261	-0.0095
4	0	4	3	1	3	3221.4542	-0.0156
3	1	3	2	0	2	3871.7394	-0.0009
4	1	4	3	1	3	4005.1811	0.0090
4	0	4	3	0	3	4052.1604	-0.0142
2	2	1	1	1	0	4732.1290	0.0057
4	1	4	3	0	3	4835.8771	0.0003
5	1	5	4	1	4	5005.4776	-0.0089
5	0	5	4	0	4	5061.1560	-0.0159
6	0	6	5	1	5	5339.5645	0.0119
5	1	5	4	0	4	5789.1852	-0.0035
3	2	1	2	1	2	5800.6252	-0.0185
7	0	7	6	1	6	6405.3827	0.0135
4	2	3	3	1	2	6696.6364	0.0102
6	1	6	5	0	5	6733.2156	0.0113
4	2	2	3	1	3	6858.9573	0.0064
3	3	1	2	2	0	7569.9370	0.0019
3	3	0	2	2	1	7570.4771	-0.0120
5	2	4	4	1	3	7659.5574	0.0094
7	1	7	6	0	6	7669.8222	-0.0025
5	2	3	4	1	4	7935.4559	0.0020
						$^{13}C_6$=$^{13}C_{10}$	
2	1	2	1	0	1	2890.4505	0.0057
3	0	3	2	0	2	3032.9208	0.0117
4	0	4	3	1	3	3210.4657	-0.0004
3	1	3	2	0	2	3863.7834	0.0058
4	1	4	3	1	3	3994.3099	0.0206
5	0	5	4	1	4	4263.7904	-0.0019
2	2	1	1	1	0	4727.3815	-0.0202
2	2	0	1	1	1	4753.6538	-0.0120
4	1	4	3	0	3	4825.1665	0.0086
5	1	5	4	1	4	4991.8949	0.0134
5	0	5	4	0	4	5047.6154	0.0000
5	1	4	4	1	3	5120.2995	-0.0112
6	0	6	5	1	5	5323.1975	-0.0051
3	2	2	2	1	1	5713.3895	0.0059
5	1	5	4	0	4	5775.7184	0.0139
3	2	1	2	1	2	5793.2681	-0.0016
4	2	3	3	1	2	6686.4276	0.0013
6	1	6	5	0	5	6716.9332	-0.0149
4	2	2	3	1	3	6848.9102	-0.0059
3	3	1	2	2	0	7563.0049	0.0157
3	3	0	2	2	1	7563.5442	0.0001
5	2	4	4	1	3	7646.5927	0.0029
7	1	7	6	0	6	7650.7758	-0.0175
						$^{13}C_7$=$^{13}C_9$	
2	1	2	1	0	1	2888.6772	0.0022
3	0	3	2	0	2	3025.2095	0.0007
4	0	4	3	1	3	3196.4205	-0.0060
3	1	3	2	0	2	3859.9243	-0.0001
4	1	4	3	1	3	3984.6418	-0.0072

Table S15. Continued.

J'	K_a'	K_c'	J''	K_a''	K_c''	Obs	Res
4	0	4	3	0	3	4031.1397	-0.0025
5	0	5	4	1	4	4246.7681	-0.0016
2	2	1	1	1	0	4731.0874	0.0046
2	2	0	1	1	1	4756.9947	0.0045
4	1	4	3	0	3	4819.3651	0.0005
5	1	5	4	1	4	4979.8646	0.0054
5	0	5	4	0	4	5034.9931	0.0009
5	1	4	4	1	3	5106.5891	-0.0032
6	0	6	5	1	5	5303.2242	0.0033
3	2	2	2	1	1	5714.8065	-0.0106
5	1	5	4	0	4	5768.0825	0.0009
3	2	1	2	1	2	5793.6213	0.0203
6	1	6	5	1	5	5974.4690	-0.0027
7	0	7	6	1	6	6363.4843	-0.0037
4	2	3	3	1	2	6685.7894	0.0046
4	2	2	3	1	3	6845.9955	0.0047
3	3	1	2	2	0	7569.7011	-0.0082
3	3	0	2	2	1	7570.2314	-0.0164
7	1	7	6	0	6	7639.6556	0.0016
5	2	4	4	1	3	7644.0484	0.0044
5	2	3	4	1	4	7916.2761	0.0039
						$^{13}C_8$	
3	1	3	2	0	2	3860.1849	0.0002
4	0	4	3	0	3	4037.9055	-0.0011
5	0	5	4	1	4	4262.4293	-0.0036
2	2	1	1	1	0	4732.4362	0.0150
4	1	4	3	0	3	4818.4043	-0.0070
5	1	5	4	1	4	4984.8746	0.0094
5	0	5	4	0	4	5042.9377	0.0001
5	1	4	4	1	3	5119.6195	-0.0045

J'	K_a'	K_c'	J''	K_a''	K_c''	Obs	Res
6	0	6	5	1	5	5322.6922	0.0027
3	2	2	2	1	1	5716.4159	0.0064
5	1	5	4	0	4	5765.3658	-0.0040
4	2	3	3	1	2	6686.8276	0.0120
6	1	6	5	0	5	6702.7338	-0.0062
4	2	2	3	1	3	6857.7311	-0.0007
3	3	1	2	2	0	7572.6313	-0.0025
3	3	0	2	2	1	7573.2249	-0.0188
7	1	7	6	0	6	7632.6052	-0.0009
5	2	4	4	1	3	7643.7149	0.0084
						$^{13}C_{12}$	
2	1	2	1	0	1	2891.0989	0.0050
3	1	3	2	0	2	3864.3054	0.0087
5	0	5	4	1	4	4284.0342	-0.0059
2	2	0	1	1	1	4754.5671	-0.0136
4	1	4	3	0	3	4824.8192	0.0179
5	1	5	4	1	4	4999.5273	0.0120
5	0	5	4	0	4	5058.4128	0.0002
3	2	2	2	1	1	5713.1802	-0.0035
3	2	1	2	1	2	5798.7228	0.0050
6	1	6	5	0	5	6713.2930	-0.0106
4	2	2	3	1	3	6860.2206	-0.0057
3	3	1	2	2	0	7562.0580	-0.0125
3	3	0	2	2	1	7562.7247	0.0193
7	1	7	6	0	6	7645.2067	-0.0072
						$^{13}C_{13}$	
2	1	2	1	0	1	2884.2923	0.0034
4	0	4	3	1	3	3208.6471	0.0103
3	1	3	2	0	2	3853.6000	-0.0010
4	0	4	3	0	3	4034.3225	-0.0072

J'	K_a'	K_c'	J''	K_a''	K_c''	Obs	Res
5	0	5	4	1	4	4262.4005	-0.0005
2	2	0	1	1	1	4749.8134	-0.0005
4	1	4	3	0	3	4810.2580	0.0019
5	1	5	4	1	4	4979.5996	0.0028
5	1	4	4	1	3	5116.2489	-0.0089
6	0	6	5	1	5	5322.1996	0.0019
5	1	5	4	0	4	5755.5232	0.0002
3	2	1	2	1	2	5789.8033	0.0110
6	1	5	5	1	4	6137.6602	-0.0021
4	2	3	3	1	2	6673.5684	0.0015
6	1	6	5	0	5	6691.1337	-0.0010
4	2	2	3	1	3	6847.0586	-0.0032
3	3	1	2	2	0	7555.5478	-0.0177
3	3	0	2	2	1	7556.2090	0.0139
5	2	4	4	1	3	7628.8438	0.0002
						$^{13}C_{14}$	
2	1	2	1	0	1	2879.3795	0.0038
3	1	3	2	0	2	3840.5022	0.0008
5	0	5	4	1	4	4189.8185	0.0144
2	2	1	1	1	0	4743.0021	0.0089
2	2	0	1	1	1	4769.2752	0.0159
4	1	4	3	0	3	4789.6576	0.0008
5	1	5	4	1	4	4930.9166	-0.0211
6	0	6	5	1	5	5237.2773	0.0074
3	2	2	2	1	1	5716.7841	0.0086
5	1	5	4	0	4	5727.9388	-0.0069
3	2	1	2	1	2	5796.6296	-0.0225
6	1	6	5	0	5	6656.8761	-0.0018
4	2	3	3	1	2	6677.6279	0.0131
4	2	2	3	1	3	6840.0316	-0.0173

Table S15. Continued.

J'	K_a'	K_c'	J''	K_a''	K_c''	Obs	Res
**7**	1	7	6	0	6	7578.3378	0.0041

Table S16. Observed frequencies and residuals (MHz) for the parent species of ATES for $J'K_a'K_c' \leftarrow J''K_a''K_c''$ transitions using the SPFIT program.

J'	K_a'	K_c'	J''	K_a''	K_c''	Obs	Res
5	0	5	4	1	4	2133.3970	0.0051
8	1	7	7	2	6	2444.1635	0.0026
3	1	3	2	0	2	2449.6128	-0.0011
11	2	9	10	3	8	2488.3683	-0.0113
6	0	6	5	1	5	2727.1987	0.0046
4	1	4	3	0	3	2999.3160	0.0015
7	0	7	6	1	6	3324.4114	0.0063
2	2	1	1	1	0	3444.1006	-0.0006
2	2	0	1	1	1	3454.8050	0.0018
5	1	5	4	0	4	3544.1227	0.0030
10	1	9	9	2	8	3681.4428	-0.0002
8	0	8	7	1	7	3924.4472	0.0026
3	2	2	2	1	1	4004.1719	-0.0013
3	2	1	2	1	2	4036.5234	0.0257
6	1	6	5	0	5	4084.3345	-0.0006
11	1	10	10	2	9	4306.8026	-0.0003
9	0	9	8	1	8	4526.6763	0.0032
4	2	3	3	1	2	4558.9411	0.0086
7	1	7	6	0	6	4620.3538	0.0042
4	2	2	3	1	3	4624.1339	0.0070
5	2	4	4	1	3	5108.3917	0.0021
10	0	10	9	1	9	5130.4082	0.0011
8	1	8	7	0	7	5152.6231	-0.0080
5	2	3	4	1	4	5218.1417	0.0079
3	3	1	2	2	0	5558.7483	0.0161
3	3	0	2	2	1	5558.8554	0.0132
6	2	5	5	1	4	5652.5697	0.0034
9	1	9	8	0	8	5681.7270	0.0046
11	0	11	10	1	10	5734.9338	-0.0052
6	2	4	5	1	5	5819.0801	0.0064
4	3	2	3	2	1	6129.1751	-0.0003
4	3	1	3	2	2	6129.7254	-0.0018
7	2	6	6	1	5	6191.4969	-0.0002
10	1	10	9	0	9	6208.2161	-0.0128
12	0	12	11	1	11	6339.5398	-0.0211
7	2	5	6	1	6	6427.6202	0.0132
5	3	3	4	2	2	6699.2019	0.0017
5	3	2	4	2	3	6700.8607	0.0009
8	2	7	7	1	6	6725.2289	-0.0025
11	1	11	10	0	10	6732.8163	0.0098
13	0	13	12	1	12	6943.5750	-0.0161
8	2	6	7	1	7	7044.4953	0.0053
9	2	8	8	1	7	7253.8287	-0.0062
12	1	12	11	0	11	7256.1369	-0.0028
6	3	4	5	2	3	7268.5044	-0.0005
6	3	3	5	2	4	7272.3874	-0.0006
14	0	14	13	1	13	7546.4003	-0.0009
4	4	0	3	3	1	7668.1619	-0.0034
4	4	1	3	3	0	7668.1619	-0.0034
9	2	7	8	1	8	7670.5601	0.0002
10	2	9	9	1	8	7777.3930	-0.0006
13	1	13	12	0	12	7778.9177	-0.0021
7	3	5	6	2	4	7836.7226	0.0117
7	3	4	6	2	5	7844.5105	0.0125
11	2	9	11	1	10	2043.2678	0.0044
10	2	8	10	1	9	2075.7497	0.0001
9	2	7	9	1	8	2109.2231	-0.0046
8	2	6	8	1	7	2142.6368	0.0011
7	2	5	7	1	6	2174.9985	0.0228
6	2	4	6	1	5	2205.3351	0.0013
4	2	2	4	1	3	2256.9489	0.0000
3	2	1	3	1	2	2276.9074	0.0018
3	2	2	3	1	3	2339.9325	0.0192
4	2	3	4	1	4	2361.2361	0.0064
5	2	4	5	1	5	2387.9431	0.0044
6	2	5	6	1	6	2420.0820	0.0029
7	2	6	7	1	7	2457.6950	0.0016
8	2	7	8	1	8	2500.8256	-0.0007
9	2	8	9	1	9	2549.5233	0.0008
10	2	9	10	1	10	2603.8239	0.0000
11	2	10	11	1	11	2663.7660	-0.0016
12	2	11	12	1	12	2729.3751	-0.0083
13	2	12	13	1	13	2800.6953	0.0045
15	3	12	15	2	13	3685.5625	-0.0006
14	3	11	14	2	12	3720.0186	-0.0055
13	3	10	13	2	11	3749.4172	-0.0005
12	3	9	12	2	10	3773.9217	0.0050
11	3	8	11	2	9	3793.8332	0.0000
10	3	7	10	2	8	3809.5853	-0.0015
9	3	6	9	2	7	3821.6677	-0.0030
8	3	5	8	2	6	3830.6178	0.0000
7	3	4	7	2	5	3836.9689	-0.0011
6	3	3	6	2	4	3841.2512	-0.0019
5	3	2	5	2	3	3843.9506	-0.0049
4	3	1	4	2	2	3845.5091	-0.0045
3	3	0	3	2	1	3846.2906	-0.0107
3	3	1	3	2	2	3846.8257	-0.0217
4	3	2	4	2	3	3847.1443	-0.0042
5	3	3	5	2	4	3847.7571	-0.0024
6	3	4	6	2	5	3848.8343	0.0014

Table S16. Continued.

J'	K_a'	K_c'	J''	K_a''	K_c''	Obs	Res
7	3	5	7	2	6	3850.5470	-0.0004
8	3	6	8	2	7	3853.1041	-0.0033
9	3	7	9	2	8	3856.7400	0.0014
10	3	8	10	2	9	3861.6850	-0.0017
11	3	9	11	2	10	3868.2132	-0.0011
12	3	10	12	2	11	3876.5979	0.0005
14	3	12	14	2	13	3900.0892	0.0053
15	3	13	15	2	14	3915.7841	0.0057
15	4	11	15	3	12	5363.5679	0.0042
14	4	10	14	3	11	5369.3092	0.0022
15	4	12	15	3	13	5376.2073	-0.0004
12	4	8	12	3	9	5377.1406	-0.0063
14	4	11	14	3	12	5377.7901	0.0012
13	4	10	13	3	11	5379.2738	0.0016
11	4	7	11	3	8	5379.6881	-0.0032
12	4	9	12	3	10	5380.6119	0.0004
10	4	6	10	3	7	5381.5658	0.0031
11	4	8	11	3	9	5381.7835	0.0051
10	4	7	10	3	8	5382.7664	0.0071
9	4	5	9	3	6	5382.9149	0.0072
9	4	6	9	3	7	5383.5634	0.0095
8	4	4	8	3	5	5383.8619	0.0138
8	4	5	8	3	6	5384.1773	0.0054
7	4	3	7	3	4	5384.4715	-0.0116
7	4	4	7	3	5	5384.6190	-0.0116
6	4	2	6	3	3	5384.8821	-0.0117
6	4	3	6	3	4	5384.9363	-0.0166
5	4	1	5	3	2	5385.1512	-0.0031
5	4	2	5	3	3	5385.1512	-0.0031
4	4	1	4	3	2	5385.2779	-0.0105
4	4	0	4	3	1	5385.2779	-0.0105
15	5	10	15	4	11	6917.3242	-0.0057
15	5	11	15	4	12	6917.6030	-0.0080
14	5	9	14	4	10	6918.9336	-0.0046
14	5	10	14	4	11	6919.1015	0.0001
13	5	8	13	4	9	6920.2258	-0.0071
13	5	9	13	4	10	6920.3256	0.0017
12	5	7	12	4	8	6921.2911	0.0055
12	5	8	12	4	9	6921.2911	0.0055
11	5	6	11	4	7	6922.0525	-0.0132
11	5	7	11	4	8	6922.1013	0.0114
10	5	6	10	4	7	6922.6895	0.0016
10	5	5	10	4	6	6922.6895	0.0016
9	5	5	9	4	6	6923.1470	0.0003
9	5	4	9	4	5	6923.1470	0.0003
8	5	4	8	4	5	6923.4793	-0.0021
8	5	3	8	4	4	6923.4793	-0.0021
7	5	3	7	4	4	6923.7133	-0.0036
7	5	2	7	4	3	6923.7133	-0.0036
6	5	2	6	4	3	6923.8704	-0.0046
6	5	1	6	4	2	6923.8704	-0.0046
5	5	0	5	4	1	6923.9663	-0.0088
5	5	1	5	4	2	6923.9663	-0.0088

Table S17. Observed frequencies and residuals (MHz) for the ^{13}C isotopologues of ATES for $J'K_a'K_c' \leftarrow J''K_a''K_c''$ transitions using the SPFIT program.

J'	K_a'	K_c'	J''	K_a''	K_c''	Obs	Res
						$^{13}C_2$	
5	3	3	4	2	2	6693.2032	-0.0064
5	3	2	4	2	3	6694.8911	0.0074
4	4	1	3	3	0	7660.8907	-0.0007
4	4	0	3	3	1	7660.8907	-0.0007
						$^{13}C_4$	
4	3	2	3	2	1	6128.2571	0.0022
4	3	1	3	2	2	6128.8031	-0.0006
5	3	3	4	2	2	6698.2179	-0.0008
4	4	0	3	3	1	7666.9764	-0.0006
4	4	1	3	3	0	7666.9764	-0.0006
						$^{13}C_5$=$^{13}C_{11}$	
3	3	1	2	2	0	5537.9806	-0.0119
3	3	0	2	2	1	5538.1068	0.0080
4	3	2	3	2	1	6107.9441	0.0056
4	3	1	3	2	2	6108.4780	0.0066
5	3	3	4	2	2	6677.4777	-0.0022
5	3	2	4	2	3	6679.0797	-0.0030
6	3	3	5	2	4	7250.0758	-0.0006
4	4	0	3	3	1	7639.2265	-0.0017
4	4	1	3	3	0	7639.2265	-0.0017
						$^{13}C_6$=$^{13}C_{10}$	
5	1	5	4	0	4	3530.8712	0.0108
3	3	1	2	2	0	5531.2441	0.0036
3	3	0	2	2	1	5531.3450	-0.0023
6	2	5	5	1	4	5628.7536	0.0016
9	1	9	8	0	8	5662.5394	-0.0046
4	3	1	3	2	2	6100.1538	0.0023
5	3	3	4	2	2	6667.5829	-0.0022
5	3	2	4	2	3	6669.2016	0.0058
6	3	4	5	2	3	7234.8678	0.0120
6	3	3	5	2	4	7238.6183	-0.0059
4	4	1	3	3	0	7630.0863	-0.0033
4	4	0	3	3	1	7630.0863	-0.0033
7	3	5	6	2	4	7801.0517	-0.0081
7	3	4	6	2	5	7808.6139	-0.0028
3						$^{13}C_7$=$^{13}C_9$	
5	1	5	4	0	4	3527.4862	0.0014
3	3	0	2	2	1	5538.7127	0.0002
4	3	2	3	2	1	6105.6075	0.0005
5	3	3	4	2	2	6672.2111	-0.0013
5	3	2	4	2	3	6673.7706	0.0047
6	3	4	5	2	3	7238.1391	-0.0053
6	3	3	5	2	4	7241.7881	0.0095
4	4	0	3	3	1	7640.6792	-0.0014
4	4	1	3	3	0	7640.6792	-0.0014
7	3	5	6	2	4	7803.0414	-0.0061
7	3	4	6	2	5	7810.3345	-0.0005
						$^{13}C_8$	
10	0	10	9	1	9	5105.8275	0.0001
3	3	0	2	2	1	5542.3626	-0.0090
5	3	3	4	2	2	6677.1358	0.0076
4	4	0	3	3	1	7645.6660	0.0040
4	4	1	3	3	0	7645.6660	0.0040
7	3	5	6	2	4	7808.9618	-0.0009
7	3	4	6	2	5	7816.9520	-0.0030
						$^{13}C_{12}$	
3	3	1	2	2	0	5524.0734	-0.0047
3	3	0	2	2	1	5524.1953	-0.0017
4	3	2	3	2	1	6091.8546	0.0007
4	4	0	3	3	1	7620.1897	0.0041
4	4	1	3	3	0	7620.1897	0.0041
7	3	4	6	2	5	7799.4944	0.0000
						$^{13}C_{13}$	
3	3	1	2	2	0	5531.5547	0.0197
3	3	0	2	2	1	5531.6462	-0.0075
4	3	2	3	2	1	6101.0089	0.0019
4	3	1	3	2	2	6101.5868	-0.0153
5	3	3	4	2	2	6670.0278	0.0005
5	3	2	4	2	3	6671.8162	-0.0014
6	3	4	5	2	3	7238.2712	0.0000
6	3	3	5	2	4	7242.4654	0.0048
4	4	1	3	3	0	7630.2839	-0.0021
4	4	0	3	3	1	7630.2839	-0.0021
						$^{13}C_{14}$	
4	1	4	3	0	3	2988.2398	0.0006
4	3	2	3	2	1	6123.3256	-0.0004
5	3	3	4	2	2	6690.0050	-0.0109
5	3	2	4	2	3	6691.6297	0.0101
4	4	1	3	3	0	7665.3342	0.0008
4	4	0	3	3	1	7665.3342	0.0008
						$^{13}C_{15}$=$^{13}C_{17}$	
5	1	5	4	0	4	3522.0638	-0.0006
3	3	1	2	2	0	5537.1386	0.0042
3	3	0	2	2	1	5537.2378	0.0012
4	3	2	3	2	1	6102.8999	-0.0006
4	3	1	3	2	2	6103.4070	-0.0055
5	3	3	4	2	2	6668.2871	0.0089
5	3	2	4	2	3	6669.8192	0.0010
6	3	3	5	2	4	7236.5878	-0.0027

Table S17. Continued.

J'	K_a'	K_c'	J''	K_a''	K_c''	Obs	Res
4	4	1	3	3	0	7638.8576	-0.0040
4	4	0	3	3	1	7638.8576	-0.0040
7	3	5	6	2	4	7796.6788	0.0012
7	3	4	6	2	5	7803.9000	-0.0021

Table S18. Observed frequencies and residuals (MHz) for DAES for $J'K_a'K_c' \leftarrow J''K_a''K_c''$ transitions using the SPFIT program.

J'	K_a'	K_c'	J''	K_a''	K_c''	Obs	Res
3	3	0	2	2	1	3902.6774	-0.0050
3	3	1	2	2	0	3902.6774	-0.0050
4	3	2	3	2	1	4173.7839	0.0082
4	3	1	3	2	2	4173.8303	0.0155
11	2	9	10	1	10	4911.1865	0.0018
7	3	4	6	2	5	4987.3455	-0.0022
8	3	6	7	2	5	5257.6302	0.0023
8	3	5	7	2	6	5258.6204	0.0037
4	4	0	3	3	1	5409.5111	-0.0108
4	4	1	3	3	0	5409.5111	-0.0108
9	3	7	8	2	6	5528.3080	-0.0003
9	3	6	8	2	7	5529.9635	0.0052
5	4	1	4	3	2	5680.6358	-0.0006
5	4	2	4	3	1	5680.6358	-0.0006
10	3	8	9	2	7	5798.8062	0.0092
6	4	2	5	3	3	5951.7422	-0.0068
6	4	3	5	3	2	5951.7422	-0.0068
11	3	9	10	2	8	6069.0493	0.0049
7	4	4	6	3	3	6222.8431	-0.0146
7	4	3	6	3	4	6222.8431	-0.0146
8	4	4	7	3	5	6493.9562	-0.0044
8	4	5	7	3	4	6493.9562	-0.0044
13	3	10	12	2	11	6616.4758	-0.0179
9	4	6	8	3	5	6765.0576	0.0029
9	4	5	8	3	6	6765.0576	0.0029
5	5	0	4	4	1	6916.3507	0.0001
5	5	1	4	4	0	6916.3507	0.0001
6	5	2	5	4	1	7187.4686	0.0031
6	5	1	5	4	2	7187.4686	0.0031
7	5	3	6	4	2	7458.5854	0.0059
7	5	2	6	4	3	7458.5854	0.0059
12	4	9	11	3	8	7578.2543	0.0028
12	4	8	11	3	9	7578.2543	0.0028
8	5	4	7	4	3	7729.6934	0.0017
8	5	3	7	4	4	7729.6934	0.0017

Table S19. Cartesian coordinates in the principal inertial axis system from the theoretical computation for AME at B3LYP-D3(BJ)/def2-TZVP.

Atom	x	y	z
C	-0.15	1.57	-0.04
H	-0.45	2.12	-0.94
H	-0.44	2.17	0.82
C	1.37	1.32	-0.04
H	1.89	2.28	-0.07
C	1.75	0.49	-1.27
H	2.83	0.31	-1.29
H	1.49	1.03	-2.19
C	1.00	-0.85	-1.23
H	1.26	-1.45	-2.11
C	-0.52	-0.59	-1.23
C	-0.90	0.24	0.00
C	-0.51	-0.52	1.27
H	-0.80	0.07	2.15
H	-1.04	-1.47	1.33
C	1.01	-0.78	1.28
H	1.28	-1.32	2.18
C	1.38	-1.61	0.04
H	2.46	-1.82	0.04
H	0.87	-2.58	0.07
C	1.76	0.56	1.23
H	1.51	1.16	2.12
H	2.84	0.39	1.25
O	-2.29	0.61	0.00
C	-3.25	-0.42	0.00
H	-3.16	-1.07	0.88
H	-4.22	0.07	0.03
H	-3.20	-1.04	-0.90
H	-1.05	-1.55	-1.22
H	-0.82	-0.06	-2.14

Table S20. Cartesian coordinates in the principal inertial axis system from the theoretical computation for ATE at B3LYP-D3(BJ)/def2-TZVP.

Atom	x	y	z
C	-0.37	-1.29	-0.59
H	0.17	-1.30	-1.54
H	-0.14	-2.22	-0.07
C	-1.88	-1.18	-0.87
H	-2.19	-2.02	-1.50
C	-2.16	0.14	-1.61
H	-3.22	0.22	-1.84
H	-1.62	0.16	-2.56
C	-1.72	1.32	-0.73
H	-1.91	2.26	-1.25
C	-0.21	1.21	-0.44
C	0.09	-0.11	0.28
C	-0.71	-0.14	1.59
H	-0.47	-1.06	2.12
H	-0.36	0.69	2.22
C	-2.21	-0.03	1.32
H	-2.75	-0.05	2.27
C	-2.50	1.29	0.59
H	-3.57	1.39	0.40
H	-2.21	2.14	1.22
C	-2.66	-1.21	0.45
H	-2.48	-2.15	0.97
H	-3.73	-1.15	0.25
O	1.45	-0.27	0.72
C	2.65	0.01	-0.02
C	2.96	1.51	-0.02
H	3.98	1.68	-0.36
H	2.29	2.07	-0.66
H	2.87	1.90	1.00
C	3.72	-0.73	0.78
H	3.51	-1.80	0.80
H	3.73	-0.37	1.81
H	4.71	-0.57	0.35
C	2.64	-0.53	-1.46
H	1.90	-0.03	-2.08
H	2.44	-1.60	-1.47
H	3.62	-0.36	-1.91
H	0.11	2.05	0.19
H	0.34	1.28	-1.38

Table S21. Cartesian coordinates in the principal inertial axis system from the theoretical computation for AMES at B3LYP-D3(BJ)/def2-TZVP.

Atom	x	y	z
**C**	-1.99	-1.62	-0.03
**H**	-1.51	-2.60	-0.05
**H**	-3.07	-1.79	-0.03
**C**	-1.59	-0.85	1.24
**H**	-1.87	-1.44	2.12
**C**	-2.29	0.51	1.27
**H**	-3.38	0.37	1.28
**H**	-2.03	1.05	2.18
**C**	-1.89	1.32	0.03
**H**	-2.39	2.29	0.05
**C**	-0.37	1.54	0.03
**C**	0.35	0.18	0.00
**C**	1.86	0.40	0.00
**O**	2.41	1.47	0.00
**O**	2.53	-0.77	0.00
**C**	3.96	-0.67	0.00
**H**	4.33	-1.69	0.00
**H**	4.31	-0.13	0.88
**H**	4.30	-0.14	-0.89
**C**	-0.07	-0.64	1.24
**H**	0.24	-0.12	2.15
**H**	0.45	-1.61	1.24
**C**	-0.07	-0.59	-1.27
**H**	0.45	-1.56	-1.30
**H**	0.24	-0.03	-2.16
**C**	-1.59	-0.80	-1.27
**H**	-1.87	-1.35	-2.18
**C**	-2.30	0.56	-1.24
**H**	-3.38	0.42	-1.26
**H**	-2.03	1.14	-2.13
**H**	-0.07	2.13	-0.83
**H**	-0.06	2.10	0.92

Table S22. Cartesian coordinates in the principal inertial axis system from the theoretical computation for ATES at B3LYP-D3(BJ)/def2-TZVP.

Atom	x	y	z
C	-3.16	0.41	1.22
H	-2.97	1.05	2.09
H	-4.22	0.14	1.25
C	-2.84	1.17	-0.07
H	-3.45	2.07	-0.13
C	-3.15	0.27	-1.28
H	-4.21	0.00	-1.30
H	-2.94	0.81	-2.21
C	-2.28	-0.99	-1.21
H	-2.49	-1.63	-2.07
C	-0.80	-0.60	-1.22
C	-0.48	0.31	-0.01
C	0.99	0.70	-0.01
O	1.39	1.84	0.00
O	1.78	-0.39	-0.02
C	3.24	-0.30	0.00
C	3.71	0.39	1.28
H	4.79	0.32	1.36
H	3.42	1.44	1.29
H	3.27	-0.10	2.15
C	3.67	-1.76	-0.01
H	4.76	-1.83	0.00
H	3.30	-2.26	-0.90
H	3.28	-2.28	0.87
C	3.74	0.42	-1.25
H	3.44	1.47	-1.25
H	3.34	-0.06	-2.14
H	4.83	0.36	-1.30
C	-1.36	1.56	-0.09
H	-1.12	2.12	-0.99
H	-1.13	2.22	0.75
C	-0.81	-0.47	1.29
H	-0.58	0.16	2.15
H	-0.18	-1.36	1.35
C	-2.30	-0.86	1.30
H	-2.51	-1.40	2.22
C	-2.60	-1.75	0.09
H	-3.65	-2.06	0.10
H	-2.00	-2.67	0.14
H	-0.17	-1.50	-1.18
H	-0.55	-0.08	-2.15

Table S23. Cartesian coordinates in the principal inertial axis system from the theoretical computation for DAES at B3LYP-D3(BJ)/def2-TZVP.

Atom	x	y	z
C	4.40	0.83	-1.26
C	3.90	1.57	-0.01
C	4.40	0.84	1.25
C	3.87	-0.60	1.26
C	4.37	-1.33	0.01
C	3.88	-0.61	-1.25
C	2.34	-0.60	-1.26
C	1.84	0.13	0.00
C	2.33	-0.59	1.26
H	1.95	-0.06	2.14
H	1.95	-1.60	1.29
O	0.39	0.28	-0.01
C	-0.44	-0.78	0.00
O	-0.08	-1.94	0.00
C	-1.89	-0.33	0.00
C	-2.18	0.54	-1.25
H	-1.51	1.40	-1.26
H	-1.97	-0.04	-2.15
C	-3.64	0.99	-1.25
C	-4.56	-0.24	-1.26
C	-4.29	-1.09	-0.01
C	-4.56	-0.26	1.25
C	-3.64	0.97	1.26
C	-3.91	1.83	0.02
H	-4.95	2.17	0.02
H	-3.28	2.71	0.02
C	-2.18	0.52	1.26
H	-1.51	1.38	1.28
H	-1.96	-0.08	2.15
H	-3.83	1.56	2.16
H	-4.39	-0.86	2.14
H	-5.61	0.06	1.27
C	-2.82	-1.55	-0.01
H	-2.61	-2.16	-0.89
H	-2.62	-2.17	0.86
H	-4.94	-1.97	-0.02
H	-4.39	-0.83	-2.16
H	-5.61	0.08	-1.27
H	-3.83	1.60	-2.14
C	2.36	1.57	-0.01
H	1.97	2.09	0.87
H	1.98	2.08	-0.89
H	1.95	-1.62	-1.27
H	1.96	-0.08	-2.14
H	4.23	-1.13	-2.14
H	4.02	-2.37	0.01
H	5.47	-1.37	0.01
H	4.22	-1.11	2.16
H	5.49	0.84	1.27
H	4.06	1.37	2.15
H	4.26	2.60	-0.01
H	4.07	1.35	-2.16
H	5.50	0.83	-1.27
